# Estimating the economic impact of canine rabies to Viet Nam 2005–2014

**DOI:** 10.1371/journal.pntd.0006866

**Published:** 2018-10-11

**Authors:** Stephanie A. Shwiff, Vienna R. Brown, Thu Trang Dao, Julie Elser, Hoang Xuan Trung, Nguyen Ngoc Tien, Nguyen Thi Huong, Nguyen Thi Thanh Huong, Arthorn Riewpaiboon, Karina Ernst, Steven Shwiff, David Payne

**Affiliations:** 1 USDA APHIS Wildlife Services, NWRC, Fort Collins, Colorado, United States of America; 2 Department of Microbiology, Immunology, and Pathology, Colorado State University, Fort Collins, Colorado, United States of America; 3 One Health Partnership, Hanoi, Viet Nam; 4 Vietnam Academy of Social Sciences, Hanoi, Viet Nam; 5 Department of Animal Health – Ministry of Agriculture and Rural Development, Hanoi, Viet Nam; 6 General Department of Preventive Medicine – Ministry of Health, Hanoi, Viet Nam; 7 National Institute of Hygiene and Epidemiology, Hanoi, Viet Nam; 8 Faculty of Pharmacy, Mahidol University, Bangkok, Thailand; 9 Department of Economics and Finance, Texas A&M - Commerce, Commerce, Texas, United States of America; 10 United Nations Development Program, International Trade and Development, Hanoi, Viet Nam; Wistar Institute, UNITED STATES

## Abstract

The global economic impact of canine rabies has been estimated by several studies. Asia bears a disproportionate burden of this zoonosis due to high levels of human deaths and rates of post-exposure prophylaxis (PEP), but low investment in preventative dog vaccination. The same factors that cause rabies to burden much of Asia are also present in Viet Nam. This study estimated the economic burden of canine rabies in a societal perspective including direct and indirect cost of rabies in dogs, livestock, and humans. Using data collected from personal interviews, published literature, published and supplementary reports, and primary data collection, we estimated the economic impact of canine rabies in Viet Nam over a ten year period (2005–2014). We incorporated the direct and indirect costs for PEP, dog vaccination efforts, livestock losses, and disability adjusted life years (DALYs) into the analysis. General findings from this analysis indicated that over the 10 year study period, the total economic impact of canine rabies was over $719 million USD. The largest portion of impacts (92%) were made up of PEP-related costs. Canine rabies created between 36,560 and 45,700 DALYs, measured in years of life lost (YLL). A total of 914 human deaths were reported over the study period. Deaths/100,000 people were 0.11, which is lower than the reported level for Asian countries. The cost per dog vaccinated was $1.75 USD. Our results indicate that canine rabies impacts in Viet Nam are consistent with the burden elsewhere reported in Asia, with large expenditures on PEP and very small investments in dog vaccination.

## Author summary

With few exceptions, canine rabies is endemic in developing countries. The mortality rate of rabies approaches 100%. However, disease is preventable via the timely administration of post-exposure prophylaxis (PEP) following a rabid animal bite. Additionally, canine rabies can be controlled in dog populations through mass vaccination. Economics drive the majority of policy and decision making and as such, a robust understanding of the cost of prevention and control methods is useful for the development and implementation of rabies management strategies. The majority of afflicted individuals are poor and reside in rural regions, where knowledge of rabies is minimal and access and affordability of PEP are limited. A greater emphasis on mass dog vaccination is a cost effective method of eliminating canine rabies at the source, which decreases the need for human PEP and reduces the burden of human mortality within vulnerable populations.

## Introduction

Canine rabies is an economically unique zoonosis, as most of its associated costs do not result from illness in the infected individual, but rather are the consequences of human deaths and efforts to prevent the disease in humans, livestock, and companion animals. This pattern of costs reflects two basic facts: the case fatality rate of rabies in humans is nearly 100%, and the disease is preventable through timely post-exposure prophylaxis (PEP) with rabies immune globulin (RIG) and multiple doses of rabies vaccine [1]. Unfortunately, in most developing countries, RIG is often unavailable [2].

Rabies exposures in humans or livestock result in economic impacts associated with vaccination or death. Because rabies patients die quickly, and there is no effective therapy, the cost of illness is relatively small, especially in the developing world. In contrast, the major direct costs arising from factors such as PEP and livestock deaths have been characterized in numerous studies [3–6]. Resultant of the fatality of rabies and the efficacy of PEP, many individuals who are at very low risk of developing the disease still seek PEP, regardless of the recommendation of health professionals [7]. In addition to the aforementioned direct costs, canine rabies also has indirect costs, including vaccination of livestock and companion animals and laboratory-based surveillance with diagnostic testing of animals suspected of rabies, both of which are beyond the scope of this manuscript [8–10].

Other impacts of rabies on the broader economy can be captured by examining changes in different sectors that result from the direct and indirect impacts of the disease [11]. Knobel and colleagues (2005) estimated global monetary expenses resulting from rabies at $695 million annually [12], while Anderson and Shwiff (2015) updated and expanded this study to include the value of human life lost for a total global estimate of approximately 69,000 lives lost annually and a global burden of $1.2 billion USD [13]. Hampson and colleagues (2015) estimated that approximately 59,000 people die globally from rabies and, consistent with other studies, the majority of canine rabies burden falls on Africa and Asia [14].

This study examined the economic impact of canine rabies in Viet Nam for a ten year period (2005–2014) using data collected from personal interviews, published literature, published and supplementary reports, and primary data collection (during a site visit in March 2016). Rabies is endemic in the Vietnamese dog population with about 100 human cases reported annually. During the study period, an average of over 400,000 PEPs were administered each year. Overall, canine rabies in Viet Nam appears consistent with the general rabies burden characterized by other Asian countries, in that a considerable amount of human deaths occur, a significant amount of PEP is administered, dog vaccination coverage is relatively low, and there is not a national comprehensive enforced dog vaccination campaign to eliminate canine rabies [6, 13, 15–16]. Most of the exposures, PEP, and rabid dogs occur in northern, more rural, provinces. In addition, many of the victims are children from poor communities, which is also consistent with canine rabies exposure worldwide [17]. In 2010, a large canine rabies outbreak occurred and 165 suspected rabid dogs were found in 17 communes of the Lao Cai province [18–19]. This resulted in a total of 156 locals being bitten and given PEP, with three deaths. In 2011, another canine rabies outbreak was identified when nine rabid dogs from five communes in three districts of the Lao Cai province were diagnosed, biting 22 locals, which resulted in one death.

Common metrics to measure the impact of canine rabies were incorporated into this analysis, including the direct and indirect costs for PEP, dog vaccination efforts, livestock losses, and disability adjusted life years (DALYs). The results from this analysis detail the level of burden created by canine rabies in Viet Nam, examine whether canine rabies impacts in Viet Nam are consistent with other Asian countries, and determine if savings can be realized by reducing the impacts of PEP. Importantly, this study focuses exclusively on canine rabies. However, wildlife species, including bats, may be a reservoir for rabies in Viet Nam, although potential impacts associated with other animals are beyond the scope of this analysis.

In Viet Nam, rabies in dogs is a notifiable disease to the Department of Animal Health, Ministry of Agriculture and Rural Development, which reports to the World Organisation for Animal Health (OIE). There is a general surveillance and monitoring program as well as a dog vaccination program. However, like many dog vaccination programs in Asian countries, rabies control through dog vaccination and management is underfunded and rabies remains a neglected disease. Identification of the current economic costs inflicted by rabies and the cost of control measures are imperative to building widespread investment and support for canine rabies elimination.

## Methods

Estimation of the economic impact of canine rabies to Viet Nam from 2005 to 2014 required coalescing information from several sources on the direct and indirect impacts of disease. Specifically, estimates of human mortality, direct and indirect medical costs, direct and indirect costs of PEP, dog vaccinations, and livestock mortality were required, along with the costs associated with each. Here we relied on estimates from numerous sources (Table 1). The National Institute of Hygiene and Epidemiology (NIHE) in Viet Nam provided data related to annual human mortality, number of PEPs administered, and the percentage of individuals that received RIG when receiving PEP. A significant amount of information for this analysis was sourced from a report entitled, “Economic analysis of a hypothetical intensified rabies prevention and control program in Vietnam” [20 –S1 Text]. This report provided an economic analysis of the impact of rabies in Viet Nam from 2008–2009, which served as a tool to advocate for greater investment in rabies prevention and control activities. Data sourced from this report include PEP vaccine administration cost/dose (excluding vaccine cost), direct non-medical plus indirect costs of PEP per case, direct and indirect medical costs of a rabid patient, indirect costs of rabid patient per case, vaccine costs, RIG costs, vaccinator or animal health worker costs, and dog owner costs (travel time, etc). All unit costs are presented in 2017 USD. We assumed that all human patients received intramuscular (IM) vaccination and that no costs were associated with adverse reactions to the vaccine. Based on information obtained from NIHE, only a small percentage of patients (9%) receive rabies immunoglobulin (RIG), which is relatively consistent with Knobel et al., 2005, who assumed that 6% of patients receive RIG in Asia. The Department of Animal Health (DAH) provided information on the total annual dog population and the number of dogs vaccinated. The Food and Agriculture Organization of the United Nations (FAO) provided the cost associated with dog vaccines and the average slaughter weight for cattle, buffalo, and pigs. The Ministry of Agriculture and Rural Development (MARD) provided annual livestock population numbers and livestock price per kilogram. To determine the rate of rabies occurrence in livestock, information was sourced from Hampson et al., 2015.

**Table 1:** Data type, values (2017 USD), availability, and sources used in this analysis.

Data type	Value (USD)	Data available	Source
**Human**			
Human population	variable	all years	World Bank
Life expectancy	variable	all years	World Bank
Number of human deaths	variable	all years	NIHE
Number of PEPs	variable	all years	NIHE
PEP vaccine administration cost/dose (excl. vaccine cost)	8		Riewpaiboon, 2010 (S1 Text)
Direct non-medical + indirect costs of PEP/case	111		Riewpaiboon, 2010 (S1 Text)
Direct medical cost of rabid patient	58		Riewpaiboon, 2010 (S1 Text)
Direct non-medical costs of rabid patient	179		Riewpaiboon, 2010 (S1 Text)
Indirect cost of rabid patient/case	976		Riewpaiboon, 2010 (S1 Text)
RIG cost	44		Riewpaiboon, 2010 (S1 Text)
Vaccine (verorab) cost	8		Riewpaiboon, 2010 (S1 Text)
% of RIG/PEP	9%		NIHE
**Canine**			
Dog population	variable	2011–2014	Dept. of Animal Health (S2 Text)
Number of dogs vaccinated	variable	2011–2014	Dept. of Animal Health (S2 Text)
Cost of dog vaccine	0.45		FAO
Vaccinators/Animal health worker costs	1.07		Riewpaiboon, 2010 (S1 Text)
Dog owner cost (travel time, etc)	0.23		Riewpaiboon, 2010 (S1 Text)
**Livestock**			
Livestock populations	variable	all years	MARD
Rate of livestock rabies	variable	all years	Hampson et al. 2015
Livestock price/kg	variable	even years	MARD
Slaughter weight	variable	even years	FAO, Livestock sector brief

### Human case data

The human population and quantity of PEP administration associated with rabies in Viet Nam during the study period is provided in Table 2. An overwhelming majority of PEP administrations in Viet Nam were resultant from exposure to a known or suspect rabies positive dog; however, rabies can be transmitted by a number of other species (e.g. bats) and this exposure would also require PEP. The non-canine related PEPs are believed to comprise a negligible number of PEP administrations annually and thus, all PEP administrations in Viet Nam are included below.

**Table 2:** Human population and PEPs administered in Viet Nam between 2005 and 2014.

Year	Human population	# of PEPs administered
2005	83,106,300	585,251
2006	84,136,800	567,173
2007	84,220,000	450,023
2008	85,120,000	380,450
2009	86,020,000	280,453
2010	86,930,000	303,150
2011	87,840,000	342,731
2012	88,770,000	400,308
2013	89,710,000	371,153
2014	90,319,600	394,979

### Dog population

The dog population in Viet Nam was estimated to range between 6 and 8 million during the study period. Reliable data regarding the dog population was only available from the DAH (S2) between 2011 and 2014 (Table 3). To derive estimates of the dog population and the number of dogs vaccinated for 2005 through 2010, we used several estimation methods. Earlier studies estimated the dog population at approximately 6 million for years prior to 2011 [21, personal communication]. Therefore, to achieve a dog population of approximately 8.5 million by 2011 we anchored 2005 (the initial study year) at 6 million and then grew the dog population at a steady rate of 0.5 million to achieve the 2011 population. Vaccination coverage is simply the share of the total dog population that is comprised of vaccinated dogs. Reliable estimates of dog vaccination coverage were only available between 2011 and 2014. To estimate vaccination coverage for 2005 through 2010, we extrapolated values with a linear time trend and applied those values to the unknown years.

**Table 3:** Dog population and canine vaccine coverage in Viet Nam between 2005 and 2014.

Year	Dog population	# of infected provinces	# of dogs vaccinated	Vaccination coverage*
2005	6,000,000		996,300	17%
2006	6,500,000		1,297,660	20%
2007	7,000,000		1,632,610	23%
2008	7,500,000	5	2,001,150	27%
2009	8,000,000	2	2,403,280	30%
2010	8,500,000	8	2,839,000	33%
2011	8,585,856	5	3,244,595	38%
2012	8,437,861	8	3,223,263	38%
2013	8,239,877	10	3,643,674	44%
2014	8,195,809	23	3,850,391	47%

*These values are rounded to the nearest whole percent.

### Livestock

One of the most critical animal species considered in this analysis is livestock. In Viet Nam, important livestock populations that are impacted by canine rabies include cattle, pigs, and buffalo. Reliable data on the prevalence of canine rabies in livestock could not be obtained through any Vietnamese source including the DAH, MARD, or NIHE. Therefore, estimates regarding the impact of canine rabies to livestock were derived from Hampson et al., 2015. Through primary data collection for the purpose of estimating the global burden of canine rabies, Hampson et al., 2015, inferred a relationship between rabies in livestock (IL) and dog vaccination coverage (VC), as presented in Eq 1.

$$IL=0.0017*{\left(1-VC\right)}^{9}$$ (1)

It follows from Eq 1 that a lower level of vaccination coverage will lead to a higher incidence of rabies in livestock. Each of the three separate livestock species are considered for this analysis. There are no data to suggest that pigs, cattle, and buffalo would be exposed with different frequencies to rabid dogs, as such, the same rate of incidence was applied across all species. Information on the annual population of each species was obtained from the MARD for the study period and vaccination coverage in dogs was obtained from DAH (Table 3). Eq 1 was used to derive the IL to determine the number of dead livestock of each type (Table 4).

**Table 4:** Incidence of canine rabies and corresponding deaths in livestock between 2005 and 2014.

Year	Rabies incidence in livestock	Pigs (# head)	# of dead pigs*	Cattle (# head)	# of dead cows*	Buffalo (# head)	# of dead buffalo*
2005	0.000332	23,421,871	7,768	5,540,700	1,838	2,922,155	969
2006	0.000229	26,855,330	6,152	6,510,794	1,492	2,921,051	669
2007	0.000156	26,560,651	4,137	6,724,703	1,047	2,996,415	467
2008	0.000104	26,701,598	2,779	6,337,746	660	2,897,734	302
2009	0.000068	27,627,729	1,885	6,103,322	416	2,886,602	197
2010	0.000044	27,373,149	1,200	5,916,251	259	2,913,388	128
2011	0.000024	27,055,900	642	5,436,600	129	2,712,000	64
2012	0.000022	26,494,000	592	5,194,200	116	2,627,800	59
2013	0.000009	26,261,400	233	5,156,700	46	2,559,500	23
2014	0.000006	26,761,600	151	5,234,300	29	2,511,900	14

*These values were rounded to the nearest whole number.

To estimate the total impacts associated with canine rabies in livestock the value of each species must be incorporated. The information regarding the average market prices associated with each livestock type was obtained from MARD. A national census is conducted every other year (even years) and the price per kilogram for meat from each species was derived from this survey. The market price for odd years was an average of the prior and subsequent even years where data were available.

### Disease burden (DALYs)

Disability-adjusted life years (DALYs) are the sum of years of life lost (YLL) and years lost due to disability (YLD) and were calculated for each study year (t). YLL_t_ was calculated by subtracting the average age of patients at death (31 years) from the life expectancy in Viet Nam (76 years) for each year of the study and multiplying by the number of reported deaths. The average age of patients at death was determined from three years of available data (2006, 2007, 2008). Ages were reported categorically, so the mean age at death was calculated by multiplying the mean age of each age category by the average number of deaths in that category over three years, summing and dividing by the average number of deaths per year. The median age at death falls between two age categories (15–24 years and 25–34 years) so is likely around 25 years. The low median age reflects the reality that rabies disproportionately affects children. As a sensitivity analysis, patients’ age at death was allowed to vary by five years to account for the categorical nature of the data (the range of all categories except the lowest and highest was ten years). Although the incubation period of rabies may be years, the time from onset of symptoms to death is just a few days. For this reason, YLD was assumed to be 0, meaning DALY = YLL.

### Cost of prevention and control measures

The cost of prevention of human rabies includes the direct cost of administering PEP (which includes RIG in 9% of cases), costs of the vaccines, and direct non-medical and indirect costs of PEP (e.g. transportation, lost time from work, accommodation and meals, etc). Any pre-exposure prophylaxis costs are assumed to be negligible. Based on data derived from other similar countries, it was assumed that each patient receiving PEP was administered three doses of vaccine (Elser et al. 2018). The total cost of rabies in humans (TC_RH_) includes the variables that are listed in Table 5 and are incorporated into Eq 2.

**Table 5:** Variable definitions, data type, and values (2017 USD) used in this analysis.

Data type	Variable	Value
**Human**		
Human population*	POP_H_	variable
Number of human deaths	Deaths	variable
Number of PEPs	PEP	variable
PEP vaccine administration cost/dose (excl. vaccine cost)	PEPcost1	8
Direct non-medical + indirect costs of PEP/case	PEPcost2	111
Direct medical cost of rabid patient	Rabid1	58
Direct non-medical cost of rabid patient	Rabid2	179
Indirect cost of rabid patient/case	Rabid3	976
RIG cost	RIGcost	44
Vaccine (verorab) cost	HumanV	8
% of RIG/PEP	9%	-
**Canine**		
Dog population*	POP_D_	variable
Number of dogs vaccinated	VD	variable
Cost of dog vaccine	DogV	0.45
Vaccinators/animal health worker costs	Worker	1.07
Dog owner cost (travel time, etc)	Owner	0.23
**Livestock**		
Livestock populations (for each livestock type i)	POP_L_^i^	variable
Rate of livestock incidence	IL	variable
Livestock price/kg (for each livestock type i)	Price_L_^i^	variable
Slaughter weight (for each livestock type i)	Wght_L_^i^	pigs = 81kg, cattle = 176kg, buffalo = 215kg

*Not used in any formula.

$${\text{TC}}_{\text{RH}}=\text{PEP}*\left(3*\text{PEPcost}1+\text{PEPcost}2+3*\text{HumanV}\right)+\left[\left(.09*\text{PEP}\right)*\text{RIGcost}\right]+\left[\text{DEATH}*\left(\text{RABID}1+\text{RABID}2+\text{RABID}3\right)\right]$$ (2)

The total cost of rabies in dogs (TC_RD_) include the vaccine, salaries for the animal health workers, and the costs that fall to the dog owners (Eq 3).

$${\text{TC}}_{\text{RD}}=\text{VD}*\left(\text{DogV}+\text{Worker}+\text{Owner}\right)$$ (3)

This study assumed that rabies only impacts three livestock species: cattle, buffalo, and pigs. The total cost of canine rabies impacts in livestock (TC_RL_) is the sum across livestock species at the applicable level of incidence for each livestock population priced at the average slaughter weight (Eq 4).

$${\text{TC}}_{\text{RL}}=\Sigma_{i=1}^{n}[(POP_{L}^{i}*IL*Price_{L}^{i}*Wght_{L}^{i})]$$ (4)

The total cost (TC) associated with rabies is the sum of the human, dog, and livestock components (Eq 5). The values were adjusted to 2017 USD and summed across all years and species.

$$\text{TC}={\text{TC}}_{\text{RH}}+{\text{TC}}_{\text{RD}}+{\text{TC}}_{\text{RL}}$$ (5)

## Results

### Estimated disease burden

Over the ten-year period between 2005 and 2014, 25,539 pigs, 6,032 cattle, and 2,892 buffalo were estimated to have died from rabies. The total cost of lost livestock exceeded $10 million (Table 6).

**Table 6:** Annual cost (in 2017 USD) of rabies impacts in livestock (pigs, cattle, and buffalo) in Viet Nam from 2005 through 2014.

	Pigs			Cattle			Buffalo		
Year	Cost/head	# dead	Total cost	Cost/head	# dead	Total cost	Cost/head	# dead	Total cost
2005	123	7,768	955,464	575	1,838	1,056,850	505	969	489,345
2006	128	6,152	787,456	621	1,492	926,532	569	669	380,661
2007	171	4,137	707,427	666	1,047	697,302	650	467	303,550
2008	213	2,779	591,927	709	660	467,940	728	302	219,856
2009	215	1,885	405,275	832	416	346,112	872	197	171,784
2010	226	1,200	271,200	985	259	255,115	1047	128	134,016
2011	274	642	175,908	1243	129	160,347	1287	64	82,368
2012	316	592	187,072	1469	116	170,404	1495	59	88,205
2013	310	233	72,230	1618	46	74,428	1769	23	40,687
2014	306	151	46,206	1771	29	51,359	2043	14	28,602
**Total**			**4,200,165**			**4,206,389**			**1,939,074**

Between 36,560 and 45,700 years of human life were lost due to rabies-caused deaths. Table 7 presents the DALYs which are equivalent to YLL as YLD is assumed to be 0.

**Table 7:** Human rabies deaths in Viet Nam and YLL or DALYs from 2005 to 2014.

		Years of life lost (YLL)		
		Age at Death		
Year	Deaths	26	31	36
2005	84	4,200	3,780	3,360
2006	82	4,100	3,690	3,280
2007	131	6,550	5,895	5,240
2008	91	4,550	4,095	3,640
2009	68	3,400	3,060	2,720
2010	78	3,900	3,510	3,120
2011	110	5,500	4,950	4,400
2012	98	4,900	4,410	3,920
2013	105	5,250	4,725	4,200
2014	67	3,350	3,015	2,680
**Total**	**914**	**45,700**	**41,130**	**36,560**

A substantial number of lives were lost over the study period and it is likely that our estimate is an underrepresentation of the disease burden, primarily resultant from underreporting of rabies-related human deaths. Many reasons exist for underreporting, including an inability to diagnose rabies from the symptoms presented and a lack of verification of rabies positivity post-mortem. Examining the number of human deaths over the study period indicates that deaths tend to trend close to the average (91.4 per year). However, 2007 was the year with the most recorded deaths and 2014 tallied the fewest.

Utilizing the data on the number of rabies deaths, it is possible to determine the costs associated with patients infected with rabies, including medical and non-medical costs (Table 8). The average cost per human death over the ten years exceeded $1,200 USD.

**Table 8:** Costs (in 2017 USD) associated with human rabies deaths in Viet Nam from 2005 to 2014.

Year	Direct medical cost of rabid patient	Direct non-medical costs of rabid patient	Indirect medical costs of rabid patient	Total cost
2005	4,872	15,036	81,984	101,892
2006	4,756	14,678	80,032	99,466
2007	7,598	23,449	127,856	158,903
2008	5,278	16,289	88,816	110,383
2009	3,944	12,172	66,368	82,484
2010	4,524	13,962	76,128	94,614
2011	6,380	19,690	107,360	133,430
2012	5,684	17,542	95,648	118,874
2013	6,090	18,795	102,480	127,365
2014	3,886	11,993	65,392	81,271
**Total**	**53,012**	**163,606**	**892,064**	**1,108,682**

### Estimated costs of prevention and control measures

For this analysis, rabies prevention is composed of two parts: prevention of rabies in dogs through vaccination and prevention of rabies in humans through PEP. During the study period, over 25 million dogs were vaccinated against rabies. After examining the costs associated with the prevention of rabies in dogs, the vaccinator/animal health worker costs composed the largest portion of total costs. Table 9 presents the costs of controlling rabies in dogs. The average cost of vaccinating a dog was $1.75 USD.

**Table 9:** Costs (in 2017 USD) of canine rabies prevention in Viet Nam between 2005 and 2014.

Year	Number of dogs vaccinated	Dog vaccine costs	Vaccinator/animal health worker costs	Dog owner costs	Total
2005	996,300	448,335	1,066,041	229,149	1,743,525
2006	1,297,660	583,947	1,388,496	298,462	2,270,905
2007	1,632,610	734,675	1,746,893	375,500	2,857,068
2008	2,001,150	900,518	2,141,231	460,265	3,502,013
2009	2,403,280	1,081,476	2,571,510	552,754	4,205,740
2010	2,839,000	1,277,550	3,037,730	652,970	4,968,250
2011	3,244,595	1,460,068	3,471,717	746,257	5,678,041
2012	3,223,263	1,450,468	3,448,891	741,350	5,640,710
2013	3,643,674	1,639,653	3,898,731	838,045	6,376,429
2014	3,850,391	1,732,676	4,119,918	885,590	6,738,184
**Total**	**25,131,923**	**11,309,365**	**26,891,157**	**5,780,342**	**43,980,864**

After examining the costs of rabies prevention in humans, it is evident that the majority of the costs are a result of direct non-medical and indirect costs of PEP. Components of direct non-medical costs include costs to the patient and family due to transportation, meals, accommodation; time lost as a result of PEP; time lost by caregivers; and indirect costs of PEP, including cost of work absence. For this analysis, it was provided by NIHE that RIG is only given to approximately 9% of individuals that received PEP. Table 10 presents the costs of rabies prevention via PEP, with the average cost per PEP at $163 USD.

**Table 10:** Costs (in 2017 USD) of canine rabies prevention in humans in Viet Nam from 2005 through 2014.

Year	Number of human PEPs	Total vaccine cost/case	RIG cost	Vaccine admin cost/case	Direct non-medical + indirect costs of PEP	Total
2005	585,251	14,046,024	2,317,594	14,046,024	64,962,861	95,372,503
2006	567,173	13,612,152	2,246,005	13,612,152	62,956,203	92,426,512
2007	450,023	10,800,552	1,782,091	10,800,552	49,952,553	73,335,748
2008	380,450	9,130,800	1,506,582	9,130,800	42,229,950	61,998,132
2009	280,453	6,730,872	1,110,594	6,730,872	31,130,283	45,702,621
2010	303,150	7,275,600	1,200,474	7,275,600	33,649,650	49,401,324
2011	342,731	8,225,544	1,357,215	8,225,544	38,043,141	55,851,444
2012	400,308	9,607,392	1,585,220	9,607,392	44,434,188	65,234,192
2013	371,153	8,907,672	1,469,766	8,907,672	41,197,983	60,483,093
2014	394,979	9,479,496	1,564,117	9,479,496	43,842,669	64,365,778
**Total**	**4,075,671**	**97,816,104**	**16,139,657**	**97,816,104**	**452,399,481**	**664,171,346**

### Total estimated average annual economic cost of rabies in Viet Nam

The total cost of rabies in Viet Nam from 2005 to 2014 was over $719 million in 2017 USD with 914 human lives lost and between 36,560 and 45,700 years of human life lost (Table 11).

**Table 11:** Total annual cost (in 2017 USD) of rabies in Viet Nam from 2005 through 2014.

Year	Total cost of rabies prevention in humans	Total cost of rabies patients	Total cost of dog vaccination	Total value of livestock lost	Total	Human deaths	Years of life lost (YLL)*
2005	95,372,503	101,892	1,743,525	2,499,858	99,717,778	84	3,780
2006	92,426,512	99,466	2,270,905	2,091,252	96,888,135	82	3,690
2007	73,335,748	158,903	2,857,068	1,709,964	78,061,682	131	5,895
2008	61,998,132	110,383	3,502,013	1,279,648	66,890,175	91	4,095
2009	45,702,621	82,484	4,205,740	924,389	50,915,234	68	3,060
2010	49,401,324	94,614	4,968,250	660,557	55,124,745	78	3,510
2011	55,851,444	133,430	5,678,041	419,226	62,082,141	110	4,950
2012	65,234,192	118,874	5,640,710	445,507	71,439,282	98	4,410
2013	60,483,093	127,365	6,376,429	186,710	67,173,597	105	4,725
2014	64,365,778	81,271	6,738,184	127,113	71,312,346	67	3,015
**Total**	**664,171,346**	**1,108,682**	**43,980,864**	**10,344,223**	**719,605,116**	**914**	**41,130**

*This column represents the mean YLL based on the average patient age at death (31 years).

## Discussion

The largest portion of rabies costs in Viet Nam are associated with PEP and only minimal expenditures are associated with dog vaccination (Table 11). Livestock losses are an insignificant portion of total costs. However, it may be the case that livestock losses are concentrated regionally and may significantly impact individual producers. Costs associated with PEP and livestock losses are potentially preventable costs or costs that would be eventually eliminated or reduced given the elimination or reduction of canine rabies impacts in Viet Nam. These costs would represent the savings associated with increased dog vaccination and decreased incidence of PEP in humans.

A substantial number of DALYs are lost, which is the result of a combination of the number of human deaths and the younger average age associated with individuals that succumb to canine rabies. Over the 10-year study period, 914 human deaths from rabies were reported. A myriad of reasons for this number of deaths have been implicated, including some individuals using traditional medicine and other homeopathic remedies as opposed to proper PEP, other individuals improperly assume that a bite from the household pet dog will not result in rabies, children often do not recognize the risk of rabies and fail to alert an adult to a dog bite, and individuals with very limited resources do not seek care as they cannot afford to do so.

Average dog vaccination coverage is less than half of the dogs in Viet Nam, which is an insufficient level of coverage to achieve canine rabies elimination. Likely the dog vaccination coverage is similar to other canine rabies endemic countries in Asia, in that coverage levels are higher in urban and suburban areas and lower in rural areas. This only exacerbates canine rabies impacts as most of the human and livestock exposures happen in rural areas as a result of a lower vaccination rate among dogs in these areas.

This study is potentially subject to limitations associated with the reliability of the data acquired. Some of the values used in this manuscript are from other publications or worldwide entities which maintain global records (ie: the FAO). However, other values are derived from supplementary reports or internal documents provided in-country. In each case, it is difficult to ascertain the accuracy of the values. Importantly, global human rabies incidence is believed to be grossly underreported with some data suggesting that the true rabies incidence in humans is upwards of 100 times greater than that reported [12]. This study uses the best available data where possible and estimates unreported values using the closest proxy. As such the results and conclusions of this work are equally reliable as other published reports on global human rabies incidence and associated impacts.

Comparing annual disease burden against prevention and control measures reveals that Viet Nam is consistent with other rabies endemic countries in Asia as more money is spent on PEP than preventing human rabies through dog vaccination. This is very evident when examining costs associated with PEP and dog vaccination. PEP costs compose 92% of the burden of rabies in Viet Nam whereas dog vaccination costs are only 6% of the costs or burden associated with the disease. Examining the costs per dog vaccinated against the cost per PEP reveals the same trend. Prevention cost per PEP is 93 times greater than the costs per dog vaccinated. Table 12 depicts the total dog vaccination costs at the actual vaccination coverage rate as compared to the dog vaccination costs at the optimal 70% dog vaccination [5]coverage rate over the study period. Attaining the 70% dog vaccination rate needed to quell canine rabies transmission between 2005 and 2014 would result in total dog vaccination expenditures of $94 million which is just 14% of the total cost associated with human rabies and rabies prevention costs. These values clearly indicate that the most cost effective control of canine rabies in humans is through coordinated veterinary public health campaigns that prevent human rabies as opposed to suppressing disease through PEP administration.

**Table 12:** Costs (in 2017 USD) associated with human rabies prevention in dogs as compared to rabies prevention and death at the actual dog vaccination coverage rate and at the recommended 70% dog vaccination coverage.

Year	Total dog vaccination costs	Total human rabies costs and rabies prevention costs	Dog vaccination as a % of human costs	Total dog vaccination costs at 70% coverage	70% dog vaccination as a % of human costs
2005	1,743,525	95,474,395	2%	7,350,000	8%
2006	2,270,905	92,525,978	2%	7,962,500	9%
2007	2,857,068	73,494,651	4%	8,575,000	12%
2008	3,502,013	62,108,515	6%	9,187,500	15%
2009	4,205,740	45,785,105	9%	9,800,000	21%
2010	4,968,250	49,495,938	10%	10,412,500	21%
2011	5,678,041	55,984,874	10%	10,517,674	19%
2012	5,640,710	65,353,066	9%	10,336,380	16%
2013	6,376,429	60,610,458	11%	10,093,849	17%
2014	6,738,184	64,447,049	10%	10,039,866	16%
**Total**	**43,980,864**	**665,280,028**	**7%**	**94,275,269**	**14%**

The results of this study indicate that canine rabies impacts in Viet Nam are consistent with how these impacts have been characterized in Asia, specifically that there are large expenditures on PEP and very small expenditures on dog vaccination. Canine rabies elimination requires a level of dog vaccination coverage that exceeds 70% [5]. To achieve this, Viet Nam should increase expenditures on dog vaccination efforts while maintaining or increasing PEP coverage. A comprehensive dog vaccination program that targets rural areas that have lower rates of vaccination coverage combined with bite prevention programs and management of free-roaming dogs will have a substantial impact on the number of bites and potential human exposures. In poorer rural areas, incentives to vaccinate dogs, such as free vaccination, may be a prudent scheme to increase participation in dog vaccination efforts.

The Ministry of Agriculture and Rural Development and the Ministry of Health crafted a report outlining a national program toward rabies control and elimination between 2017 and 2021 [22]. This document summarizes the dog vaccination coverage goals which have been categorized for urban areas, lowland, midland and mountainous regions, and rural and remote areas. This report indicates that future dog vaccination coverage targets will increase over time across all regions. However, the highest dog vaccination coverage rates are projected for 2021. Challenges to obtain high levels of dog vaccination coverage may include the prevalence of inaccessible dogs, the inability or unwillingness of owners to bring dogs in for vaccination, the lack of information about dog populations, the lack of canine rabies surveillance and diagnostic capabilities, and insufficient resources from veterinary services [23]. Nevertheless, the benefits of eliminating the disease are tremendous.

## Supporting information

S1 Text The Riewpaiboon 2010 report, entitled ‘Economic analysis of a hypothetical intensified rabies prevention and control program in Vietnam’ is included as Table 8 provides all the values that were used in our manuscript that were derived from this report. The conversion rate used from Vietnamese dong (VND) to USD was from 2009 (17,801 VND/$1 USD); the values were then grown to 2017 USD.
(DOC)

S2 Text The data provided in-country by the Department of Animal Health, entitled ‘Canine rabies data’ is included as it provides information on the dog population and vaccination coverage. (DOCX)
