# Supplementary material for: Estimating the economic impact of canine rabies to Viet Nam 2005–2014
Source: PLoS Negl Trop Dis. 2018 Oct 11;12(10):e0006866. doi: 10.1371/journal.pntd.0006866 (PMC6199002; doi:10.1371/journal.pntd.0006866)
Supplement: S1 Text — The conversion rate used from Vietnamese dong (VND) to USD was from 2009 (17,801 VND/$1 USD); the values were then grown to 2017 USD. (DOC) [file pntd.0006866.s001.doc]

# Economic analysis of a hypothetical intensified rabies prevention and control program in Vietnam

**1. PURPOSE OF THE MISSION**

**General purpose:** To undertake an economic analysis of the impact of rabies in Vietnam, to be used as an advocacy tool for greater investment in rabies prevention and control activities.

**Specific objectives:**

1. To assess the current rabies situation in Vietnam, including burden of disease, dog vaccination coverage, costs associated with dog bite injuries, and human post-exposure prophylaxis (PEP).

2. To incorporate the recommended rabies interventions (i.e. improved dog vaccination coverage, better screening of dog-bite victims before administering post-exposure prophylaxis, increased proportion of post-exposure prophylaxis administered intradermally, improved disease surveillance) into cost-effectiveness and cost-benefit analyses.

**2. BACKGROUND**

Rabies is a significant public health problem in Asia and Africa, despite the existence of safe, effective vaccines for both human and veterinary use. There are an estimated 55,000 deaths per year and 1.78 million disability-adjusted life years (DALYs) lost due to morbidity and mortality related to rabies. The annual global economic burden of rabies is estimated to be US$ 583.5 million.^1^ There are several published economic evaluations of rabies prevention and control programs^2-7^.

In 2007, the ASEAN Plus Three member countries issued a Call for Action for rabies elimination from the region by 2020. Vietnam has committed to leading this initiative. In May 2009, the National Institute of Hygiene and Epidemiology (NIHE) in Vietnam hosted an ASEAN Plus Three workshop on advocacy for rabies policy, with a follow-up workshop scheduled in September 2009. The aim of these workshops is to develop a regional strategy for advocating for greater political commitment, resources, and funding for rabies. The proposed economic analysis of rabies prevention and control in Vietnam will serve as a practical example of an advocacy tool to ASEAN member countries.

In Vietnam, a Rabies National Control Plan has been drafted but has not yet been officially approved. The plan broadly describes activities and associated funding for rabies prevention and control. Currently, the national budget for rabies is small ($10,000-$20,000/year). It is uncertain how funding and resources for the proposed activities will be sourced; consequently, the proposed economic evaluation will provide valuable guidance to policy-makers in the decision-making process.

**3. ACTIVITIES AND FINDINGS**

Activities undertaken during 3-15 May 2009 and 9-16 June 2009 included a literature review, assessment of the current rabies situation and proposed interventions, data collection, data management and analyses. The literature review focused on the epidemiology of rabies in dogs and humans and the effects of various interventions in terms of morbidity, mortality, and economics. Data collection was composed of primary and secondary data collection. Primary data collected included costs associated with dog bite injuries, PEP, human cases of rabies, dog vaccination, laboratory investigation of dog specimens and social mobilization including information, education, and communication (IEC) approaches. These data were collected from preventive medicine centers, hospitals, and animal health offices in greater Hanoi and Thai Nguyen province (see Figure 1). Thai Nguyen province was selected for several reasons: its proximity to Hanoi and, thus, ease of travel; ongoing difficulties in reducing the incidence of human rabies cases and improving dog vaccination coverage; high numbers of patients receiving PEP; and recent human deaths from rabies. Some primary data was also collected in Ho Chi Minh City (HCMC) by a previous WHO International Consultant, Dr. Katie Hampson, in March 2009.

Secondary data included statistics related to PEP patients and human cases of rabies, collected from the National Rabies Control Program based at NIHE. Reference values, e.g. population, GDP, and income were collected from the Handbook of the General Statistics Office of Vietnam.^8^

Figure 1: Primary data collection


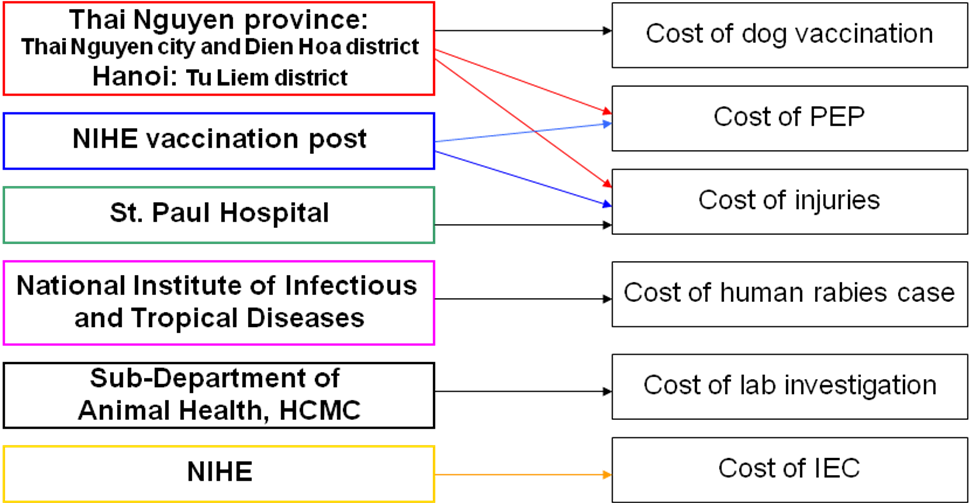


Findings from activities aforementioned are detailed below.

**1.** **Literature review**

**1.1 Burden of disease**

Rabies accounts for 1% of global mortality due to infectious diseases**.**^5^ The burden of rabies can also be estimated based on disability-adjusted life years (DALYs). Coleman et al estimated 35,000 deaths globally each year equating to approximately 1.16 million DALYs.^9^ This estimation covered only years of life lost and did not include years lived with disability due to animal bite injuries or adverse events from PEP. In 2005, Knobel et al estimated the number of deaths in Asia and Africa as 55,270 deaths.^1^ The estimated cost including PEP and costs associated with canine rabies control was US$583.5 million or 9.5 million DALYs. The DALYs covered death and disability due to nerve tissue vaccine adverse events but it was assumed that there was no disability caused by PEP itself. Disability weighted for neurological adverse events from Semple nerve tissue vaccine was 0.631. Durations of event were 1 day, 8 days, and 1 year for minimum, most likely, and maximum, respectively. This was assumed to be equal in severity to an injured spinal cord. Disability weight for suckling-mouse brain neurological adverse events was 0.725 for 200 days. This was assumed to equal in severity to bacterial meningitis. In terms of economic burden, Meltzer and Rupprecht reviewed and published an assessment of the economics of prevention and control of rabies in 1998.^10, 11^

There have also been further economic studies in subsequent years^12-15^, with differing cost descriptions. Based on findings from a thorough literature review as well as consideration of the theoretical frame of cost of illness^16^ and the WHO guidelines for estimating costs of vaccination^17^, the cost of rabies from a societal perspective can be best summarized by defining the costs within the human sector and within the animal sector. This covers both public costs (from providers such as vaccine posts and hospitals) and private costs (costs to patient, family, dog owner). The costs can be defined as an average cost or as a marginal cost of the additional interventions or outputs. The full scope of costs associated with rabies are presented below in Tables 1 and 2.

Table 1: Costs within the human health sector.

| 1.1 | Administrative activities; program management; social mobilization (including IEC) |
| --- | --- |
| 1.2 | Surveillance and epidemiology (including laboratory investigation and coordination) |
| 1.3 | Research/development of vaccine; research into prevention and control programs |
| 1.4 | Treatment of dog bite injuries |
| 1.5 | Pre-exposure prophylaxis including adverse events of rabies vaccine |
| 1.6 | PEP including adverse events of rabies vaccine and rabies immunoglobulin (RIG) |
| 1.7 | Human cases of rabies |

*Note*: The basic cost composition is labor, material, and capital costs. Items 1.1-1.3 relate to health care programs. To calculate the costs, the cost per each individual activity is computed and then added together to reflect the cost of the whole program. The items listed in 1.4-1.7 are costs of illness studies and are composed of direct medical costs or direct treatment costs (rabies vaccine; rabies immunoglobulin; RIG; other medications; diagnostic procedures and other medical services received from all health facilities), direct non-medical costs (costs to the patient and family due to transportation, meals, accommodation; time lost as a result of treatment; time lost by caregivers), and indirect costs (cost of work absence and death due to rabies).

Table 2: Costs within the animal health sector.

| 1.1 | Vaccination (acquisition cost of the vaccine, vaccine delivery cost, cost of IEC activities and campaigns, and transportation/time costs of dog owners) |
| --- | --- |
| 1.2 | Administrative activities; program management; social mobilization |
| 1.3 | Surveillance and epidemiology (including laboratory investigation and coordination) |
| 1.4 | Research/development of vaccine; research into prevention and control programs |
| 1.5 | Population control (e.g. sterilization, elimination of stray dogs) |

*Note:* Cost compositions are similar to those of the human sector, except that these activities relate to animal health programs. Each activity covers labor, material and capital costs.

**1.2 Effectiveness of rabies prevention and control interventions**

There are several published studies demonstrating the impact of intensified rabies prevention and control strategies.

*Africa*

Success of dog rabies control by mass vaccination has been demonstrated in many countries.^18^ An annual dog vaccination campaign conducted in Tanzania during 1996-2001 indicated that achieving vaccination coverage of 60-70% of dogs was sufficient to control rabies with the incidence of dog rabies declining by 97% and the demand for PEP also decreasing.^19^ Another study in Tanzania focused on strategies to increase dog vaccination coverage. The vaccination strategies trialed were central-point vaccination posts, house-to-house visits, and community-based animal health workers. Most of the strategies achieved at least 80% vaccination coverage.^20^ In Chad, cost subsidies for dog vaccine was indicated as a determining factor in achieving goals of high vaccination coverage.^21, 22^

*India*

A dog sterilization and vaccination program in Jaipur, India conducted over an 8 year period resulted in a decrease from 10 cases of human rabies per year to no cases.^23^

*Thailand*

A five-year program in a province of Thailand was implemented between 1996 and 2001. The intervention was composed of: increasing accessibility to PEP, intensifying documentation of PEP, raising public awareness by delivering IEC campaigns through schools and via television and newspapers, reducing canine rabies by implementing dog vaccination and sterilization programs, increasing cooperation between the provincial levels of the Ministries of Public Health, Agriculture and Education, and assessing the impact of the program through intensified follow-up of patients exposed to suspected and laboratory-confirmed rabid dogs. After two human deaths occurring during the first two years of the program, there were no deaths recorded in the subsequent three years of the program.^24^

**1.3 Economic evaluation of prevention and control programs**

There are several published studies concerned with undertaking economic evaluation of rabies prevention and control programs. A study in the Philippines compared costs and benefits if rabies had been eliminated in 1988. Costs of the prevention and control programs were composed of dog vaccination and population management (including controls over dog importation), laboratory diagnostics, PEP of dog bite victims, and surveillance and research. Benefits were defined as a discontinuation of expenditure for prevention of rabies in humans and animals and additional earnings associated with a decreased number of human deaths due to the disease. During a hypothetical study period of 25 years, with a dog vaccination coverage level of 60%, the benefits recouped the program costs within 4.1 years. This period was slightly longer when substituted with 80% vaccine coverage levels.

In 1990, Bogel and Meslin^6^ compared cost and effectiveness of three strategies of rabies control: intensifying and modernizing PEP (A), controlling the disease in the animal reservoir (B), and the combination of the two (A+B). The time horizon of the analysis was divided into three phases, i.e. attack phase of two years, consolidation phase of four years, and maintenance phase of four years. Effects of the intervention were reductions in PEP costs and in deaths with a 50% reduction of PEP costs expected to occur in the fifth year. From the seventh year, PEP costs were expected to be 5% of the initial value. The initial mortality rate was estimated as 6-15 deaths per million inhabitants per year. Strategy A was expected to reduce the number of deaths to 3 deaths per million inhabitants per year. Strategy B was expected to reduce number of deaths to 20% of the initial rate during the first two years, then to 0 deaths in the third year. The combined strategy was expected to reduce the number of deaths to 0 in the second year, while the first year was similar to that of the strategy B (20% of the initial rate). Across the three strategies, cost per death avoided was in the range of US$4,300-$53,330.

In 2003, cost and benefits of a hypothetical intensified dog control program achieving over 80% dog vaccination coverage were assessed in Thailand. The costs included were those associated with dog vaccination, PEP, laboratory investigation of suspect dog specimens, and social mobilization. Benefits were defined as productivity gained due to death averted. During the 3 year time horizon of the analysis, the number of deaths in the first two years was expected to be 20% of the pre-intervention period and no deaths were forecasted in the third year. No effect on the rate of PEP usage was expected and the incremental benefit cost ratio was 2.04.^4^

Recently, Dhankhar et al^2^ investigated cost effectiveness of rabies PEP in USA in 2008. The costs included were the acquisition cost of rabies vaccine and RIG, the treatment costs of vaccination (excluding the cost of treating injuries), and patient costs relating to travel, lost wages, additional medicines, and productivity loss due to death. Based on expert opinion of the probabilities of rabies transmission to a person, PEP administration represented a cost saving from a societal perspective if the risk of getting rabies of a patient was greater than 0.7%. For lower risks of transmission in various scenarios, the cost per life saved ranged from US$500,000 to billions of USD.

In summary, there are several published cost studies but few actual economic evaluations of rabies prevention and control programs comparing differences between interventions and associated costs. Estimated effects of interventions also vary between studies and costs differ between countries which places limitations on the generalisability of such studies.

**2. Current rabies situation in Vietnam**
The dog population in Vietnam is estimated to be approximately 6 million^25^ (in between 5-7 million).^26^ However, accurate census data is lacking. Dog vaccination coverage rate is estimated to be >90% in urban areas, 70% in lowland areas, 40% in rural highland areas, and 30% in rural areas of mountainous provinces.^25^

Information collected relating to population size, numbers of PEP patients, and numbers and rates of human deaths is presented in Table 3. Mortality rates classified by regions and age groups are presented in Tables 4 and 5.

**Table 3: Current situation of rabies in Vietnam.**

| Year | Population | PEP patients | Death cases |
| --- | --- | --- | --- |
| 2000 | 77,635,400 | 568,166 | 90 |
| 2001 | 78,685,800 | 552,653 | 65 |
| 2002 | 79,727,400 | 637,185 | 47 |
| 2003 | 80,902,400 | 635,815 | 34 |
| 2004 | 82,031,700 | 607,720 | 84 |
| 2005 | 83,106,300 | 585,251 | 84 |
| 2006 | 84,136,800 | 567,173 | 82 |
| 2007 | 85,171,700 | 450,023 | 131 |
| Average |  |  |  |

Table 4: Death cases by region.

| Region | Number of province | Average annual death during 2004-8 | Average death per million population in 2008 |
| --- | --- | --- | --- |
| North East | 11 | 47.80 | 5.09 |
| Central Highlands | 5 | 11.40 | 2.28 |
| South Central Coast | 6 | 6.60 | 0.92 |
| Mekong River Delta | 13 | 12.00 | 0.69 |
| South East | 8 | 6.20 | 0.44 |
| North Central Coast | 6 | 4.40 | 0.41 |
| Red River Delta | 11 | 6.65 | 0.36 |
| North West | 4 | 0.20 | 0.08 |
| TOTAL | 64 | 95.25 | 1.12 |

Table 5: Number of deaths classified by age groups.

|  | Number of death | | |
| --- | --- | --- | --- |
| Age | 2006 | 2007 | 2008 |
| 0-14 yrs | 20 | 18 | 23 |
| 15-24 yrs | 9 | 7 | 14 |
| 25-34 yrs | 15 | 13 | 9 |
| 35-44 yrs | 9 | 13 | 12 |
| 45-54 yrs | 9 | 18 | 5 |
| 55-64 yrs | 3 | 6 | 5 |
| >65 yrs | 4 | 7 | 4 |
| Total | 69 | 82 | 72 |

**3. Proposed rabies prevention and control program in Vietnam**

The ultimate purpose of intensifying the rabies prevention and control activities in Vietnam is to prevent human deaths from disease. Three key objectives, and necessary activities, are listed below:

1. To increase dog vaccination coverage to 70% and maintain this for 10 years

- IEC and workshops in communities to improve knowledge and empower communities (social mobilization)
- Cost-sharing mechanism (government/owner) in poor areas
- Improved disease surveillance in dogs

2. To reduce costs of PET

- Reduce exposure to rabies via IEC, improved vaccine coverage in dogs, and improved disease surveillance in dogs and humans
- Reduce unnecessary PEP administration by improving disease surveillance in dogs, improved vaccine coverage in dogs, and better training of health care workers (better patient screening)
- Reduce the cost of each PEP course by training health care workers to administer a greater proportion of PEP via the intradermal (ID) route

3. Improved disease surveillance via increased investigation of suspected rabid dogs with laboratory diagnostics

**3.1 Assumptions used in the economic analysis**

The current level of dog vaccination coverage is lowest in highland and mountainous provinces, with an estimated 35% coverage. The scope of this economic analysis is, therefore, focused on these provinces where there is an obvious need for greater investment in rabies prevention and control activities.

A vaccine cost subsidy program and strengthened IEC activities are, together, proposed to achieve 70% dog vaccination coverage. Based on the findings of the literature review, it can be assumed that the impact of achieving a dog vaccination coverage level of 70% on human mortality rates will lead to a gradual reduction to reach zero deaths by the end of the fifth year of the program, maintaining this status until the tenth year. This a conservative estimate when compared to that of Bogel and Meslin or the hypothetical Thai study ^4, 6^.

The combination of strengthened disease surveillance and training of health care workers is assumed to result in a decrease in PEP administration. The incidence of PEP cases can be assumed to gradually decrease to 50% of the initial figure by the end of the fifth year, then to 25% by the end of the tenth year. Again, this a conservative estimate when compared to that of Bogel and Meslin^6^ however, it should also be noted that it is unusual for intensive rabies programs to result in a reduction in requirement for PEP in the short-term, with the Tanzanian study as an exception^19^.

The training of health care workers is also assumed to increase the proportion of PEP administered by the intradermal route. Based on a review conducted by Dr. Katie Hampson in March 2009, a previous WHO International Consultant, 10% of all PEP patients in Vietnam are treated at vaccine posts receiving a low number of patients (i.e. less than 18 new patients per month). It is likely that the IM route of administration for PEP will be preferred by staff at these posts, due to the risk of vaccine wastage with ID administration and inability to recoup costs given the full cost-recovery model currently implemented in Vietnam. Taking this into account, it is assumed that an intervention focusing on training health care workers will be unlikely to increase the proportion of PEP administered via the ID route beyond 90%. Accurate and complete data is not available relating to the administration routes of PEP; however, based on available data, it is unlikely that the proportion of PEP administered via the ID route is greater than 50%. We can assume a conservative estimate of increasing this proportion to 80%.

As per WHO guidelines for the declaration of rabies-free zones, the number of suspected rabid dogs tested for rabies should be at least 0.015% of estimated dog population. This figure is assumed to be constant throughout the entire 10 years.

Costs associated with IEC activities, training courses, and program management are based on those dictated by the Ministry of Health and Ministry of Agriculture and Rural Development in the proposed, but not yet official, National Control Plan for Rabies. ^27^

In relation to the proposed cost-sharing mechanism (government/owner) in rural, poor areas, the total costs are the same from a societal perspective, regardless of the contribution of each party.

In this economic analysis, the time horizon of the analysis is proposed as 10 years, in keeping with the ASEAN Plus 3 goal to eliminate rabies from the region by 2010. It is considered that there are two choices regarding rabies prevention and control programs - to either maintain the current program or to implement the proposed intensified program. The proposed program will be economically evaluated and compared to the current program. The conceptual framework of the study is composed of a decision tree model including proposed interventions. The tree diagram of the current program is similar to that of the proposed program, but different in the prevention and control interventions. The latter is removed from the model shown in Figure 2 due to space limitations.

Figure 2: Conceptual framework of the economic analysis.

**4.** **Data collection and management**

**4.1 Estimating costs in the human sector**

Sources of data collected during the consultancy are presented below, according to the structure shown in Table 1 in Section 1.1.

**4.1.1 Costs of program management; IEC, training and social mobilization**

The cost of year-round IEC activities as a social mobilization tool and costs associated with providing training to health care workers in relation to patient screening and ID PEP administration could not be directly measured. Consequently, these costs were estimated from the budget allocation in the proposed National Control Plan.

**4.1.2 Surveillance and epidemiology costs**

Surveillance and epidemiology costs are composed of laboratory investigation of dog specimens and dissemination of surveillance information. The cost of laboratory investigation of dog specimens is based on investigation fees and the estimated cost of specimen transportation. The cost of dissemination of surveillance information is covered by the annual budget for management of rabies prevention and control programs and the cost of laboratory tests of dog specimens.

**4.1.3 Costs associated with research and development**

Costs associated with research and development of vaccine and research into prevention and control programs are not included in this study because they are relatively small and there are limitations in data collection. Note that some (but not all) of the RIG used in Vietnam is manufactured locally, whereas all Verorab rabies vaccine is imported.

**4.1.4 Cost of dog bite injuries**

Information relating to direct medical costs associated with dog bite injuries (medications, diagnostic procedures, and other medical services received from health facilities), was collected by interviewing three physicians from the vaccination post at NIHE, St. Paul Hospital in Hanoi, and the Dien Hoa district hospital in Thai Nguyen province. Direct non-medical costs and indirect costs were estimated from data obtained by interviewing PEP patients at NIHE and in Thai Nguyen province.

**4.1.5 Cost of pre-exposure prophylaxis**

Pre-exposure prophylaxis is not included in this model, as the vast majority of the at-risk population in Vietnam, including animal health workers, do not receive pre-exposure vaccination.

**4.1.6 Cost of PEP**

PEP is composed of direct medical costs, including treatment or vaccination costs (rabies vaccine, RIG), and direct non-medical costs (cost to the patient and family due to transportation, meals, accommodation, time lost as a result of treatment, time lost by caregivers). Vaccination costs were estimated by interviewing staff at three vaccine posts, i.e. Tu Liem district, Hanoi; Thai Nguyen city; and Dien Hoa district in Thai Nguyen province. Direct non-medical costs were obtained by patient interviews at the aforementioned vaccine posts. Adverse events from the vaccine (Verorab) and RIG (Favirab) are assumed to be none, due to high reported vaccine safety.^28, 29^

**4.1.7 Cost of human cases of rabies**

Direct medical costs associated with human cases of rabies were collected from the medical records of 7 patients hospitalized during 2008 at the National Institute of Infectious and Tropical Diseases in Hanoi. Direct non-medical costs and indirect costs were estimated based on treatment of these patients. Productivity loss due to death was calculated employing the human capital approach^30^. Per capita GDP and average daily income in Vietnam were included in the calculation.^8^

**4.2 Estimating costs in the animal sector**

Sources of data are presented below, according to the structure shown in Table 2 in Section 1.1.

**4.2.1 Costs of dog vaccination**

Animal health staff in Tu Liem district in Hanoi and Dien Hoa district in Thai Nguyen were interviewed to obtain information about the acquisition cost of the vaccine, vaccine delivery cost, cost of the campaign, and transportation and time costs of dog owners. Costs of vaccine delivery included the cold chain system as well as salaries, materials and equipment. Estimation of transportation and time costs of dog owners were based on experiences of the animal health staff, rather than through directly interviewing dog owners.

**4.2.2 Costs of IEC campaigns and management costs**

Costs of IEC campaigns and management costs of a dog vaccination could not be directly measured. Consequently, these costs were estimated from the budget allocation in the proposed National Control Plan.

**4.2.3 Cost of laboratory investigation**

Costs associated with laboratory investigation of specimens from suspected rabid dogs were previously collected from the Department of Animal Health in HCMC.

**4.2.4 Cost of dog vaccine research and development**

All vaccine is imported, so there are no costs associated with vaccine research and development.

**4.2.5 Cost of dog population control**

Population control is not included within the scope of this study. Cultural practices relating to dog meat consumption may impact dog population dynamics.

**4.3 Cost estimation of the proposed program**

Currently, the Vietnamese government invests 3-5 billion VND annually towards the prevention and control of rabies, including the following activities:

- Staff salaries
- Training and workshops for human health and animal health workers
- IEC activities
- Support for research and development of rabies vaccines and RIG

In addition, each province allocates an average of 70-80 million VND annually from their respective provincial budgets.

The budget of the proposed intensified program in this economic analysis is estimated based on the budget allocation in the National Control Plan for Rabies, proposed by the Ministry of Agriculture and Rural Development and the Ministry of Health ^27^**.** The budget required for the first 5 years of an intensified program is composed of the following:

- State budget 73,921.24 million VND
- Local budget 14,800 million VND
- International funding 1,500 million VND

This budget covers the following activities: training courses/workshops, IEC activities, disease surveillance, research and development, and program administration.

**4.3** **Parameters** **for analysis**

Table 4 below shows the parameters to be used in the economic analysis, based on the cost data collected during the consultancy and findings from the literature review.

Table 6: Review of parameters.

| **Parameter** | **Review** |
| --- | --- |
|
| **Epidemiology /Reference value** |  |
| Population | 86,210,800 in 2008^8^ |
| Average population growth rate | 1.32%^8^ |
| Dog/inhabitant ratio | In Vietnam: 6 million^25^ (range: 5-7 million)^26^  for 86,210,800 people  Internationally: varying from 1:5, 1:10, 1:15 in the Phillines^7^, 1:8 in 14 developing countries of Africa, Asia, South and Central America^6^ |
| Dog vaccination/ coverage rate (%) | Currently in Vietnam: >90% in urban areas, 70% in low land area, 40% in rural areas of highland provinces, 30% in rural areas of mountainous provinces^25^  Intervention: 70%^31^,75%^6^, 80%^4, 31^ |
| Life expectancy (target to 2010) | 72 years^32^ |
| GDP per capita 2008 (x 1000VND) | 17,141^8^ |
| Average annual growth of GDP per capita (current price) | 0.14^8^ |
| Daily income (x1000VND) | 88.38^8^ |
| Average annual growth of income | 17.72^8^ |
| Discount rate | 8.94% and 15%^7^, 10%^6^, 3% and 5%^4^, 3%^2^ |
| **Effect** |  |
| Duration of the intervention | 3 years in Thai study^4^, 10 years by study of Bogel and Meslin^6^ and 25 years by a study in the Philippines^7^ |
| Effect of surveillance and training on PEP | 50%, 33%, 25% and 5% of initial PEP incidence is achieved by Year 3, 4, 5 and after Year 5, respectively (Bogel and Meslin)^6, 33^  However, in Thailand, dog vaccination coverage has reached approximately 65%-70%, yet the PEP usage has not decreased.^34^ |
| Effect of training on proportion of ID_PEP | Based on review of Dr Hampson^35^ |
| Effect of 70% coverage on mortality rate | A hypothetical study in Thailand estimated the number of deaths during the 1^st^ and 2^nd^ years of intervention as 20% of the pre-intervention rate, with no deaths occurring during the 3^rd^ and subsequent years.^4^ Bogel and Meslin proposed that “the number of human deaths would decrease quickly and reach zero level after 2-3 years, provided that a well planned comprehensive canine rabies control program is correctly implemented.” ^6^ |
| Death cases, stratified by age and provinces | Age and province-specific death rates were reviewed. |
| **Dog vaccination cost** |  |
| Rabisin (cell culture, France) | 3,500VND/dose (10-dose vial); 5,000VND/dose (single dose vial) |
| Dog vaccine administration cost | Direct cost measurement |
| Dog owner cost (travel, working time) | Direct cost measurement |
| Wastage rate of Rabisin | Assumption |
| **PET cost** |  |
| Verorab (purified vero cell culture; Sanofi Pasteur) | 148,000VND/0.5 ml labeled vial; 120,000VND/0.5 ml unlabelled vial |
| RIG cost/ dose | 600,000-700,000VND/dose (NB: one dose = one course) |
| RIG/PEP case(%) | 50% ^6^ |
| Average volume of vaccine used via IM route/ PEP case | Assumption (accounting for the fact that the full IM course will not be received by all cases) |
| Average volume of vaccine used via ID route/ PEP case | Assumption (accounting for the fact that the full ID course will not be received by all cases) |
| ID case/ PEP case (%)  (i.e. proportion of PEP courses administered via the ID route) | Statistics Jan-Apr 2009 |
| Treatment costs of dog bite injuries | Direct cost measurement |
| Direct non-medical and indirect costs/PEP case | Direct cost measurement |
| Treatment costs/ human rabies case | Direct cost measurement |
| Direct non-medical costs/human rabies case | Direct cost measurement |
| Indirect costs/human rabies case (i.e. death) | Direct cost measurement |
| Waste rate of Verorab | Assumption. |
| Assumption | Assumption |
| **Surveillance** |  |
| Proportion of dog population investigated for rabies by laboratory testing | Current: approximately 40 specimens/ 6 million dog  To declare a rabies-free country, disease surveillance must involve testing of 0.01-0.02% of the estimated dog population, with negative results.^31^ |
| Cost of investigation of suspected rabid dogs | The cost of collecting sample is about 100,000 VND/test. A rabies test is 350,000 VND. |

**5 Data Analysis**

Due to an inability to collect all necessary data during the consultancy, analysis could not be performed. However, an approach to data analysis is proposed below.

**5.1 Effectiveness of rabies prevention and control program**

Outcomes to be measured are deaths averted, disability-adjusted life year (DALYs) averted, and costs averted (savings due to the proposed program). DALYs for a disease or health condition are calculated as the sum of the Years of Life Lost (YLL) due to premature mortality in the population and the Years Lost due to Disability (YLD) for incident cases of the health condition.^36^

DALY = YLL + YLD

The YLL basically correspond to the number of deaths multiplied by the standard life expectancy at the age at which death occurs. The basic formula for YLL (without yet including other social preferences discussed below), is as follows for a given cause, age and sex:

YLL = N x L

where:

N = number of deaths

L = standard life expectancy at age of death in years

Because YLL measures the incident stream of lost years of life due to deaths, an incidence perspective is also taken for the calculation of YLD. To estimate YLD for a particular cause in a particular time period, the number of incident cases in that period is multiplied by the average duration of the disease and a weight factor that reflects the severity of the disease on a scale from 0 (perfect health) to 1 (dead). The basic formula for YLD is the following (again, without applying social preferences):

YLD = I x DW x L

where:

I = number of incident cases

DW = disability weight

L = average duration of the case until remission or death (years)

Based on a conservative analysis, disability from dog bite injuries is not accounted for in this analysis.^9^ Neurological complications and other adverse events relating to the administration of Verorab vaccine and RIG in Vietnam are assumed to be negligible, due to high reported vaccine safety^28, 29^.

**5.2 Cost-benefit and cost-effectiveness analyses**

The study is designed as a modeling of cost-benefit analysis (CBA) and cost-effectiveness analysis (CEA)^37, 38^ from a societal perspective. The intervention is the proposed intensified rabies program.

Based on a literature review, the time horizon of the analysis is proposed as 10 years with a 3% discount rate of cost and outcome.^39^ This is also in keeping with the ASEAN Plus Three goal to eliminate rabies from the region by 2020.

Net present value for CBA

Net present value = (COIc-COIp)-(CPCp-CPCc)

Where: COIc = cost of illness; rabies under the current situation

COIp = cost of illness; rabies under the proposed program

CPCc = cost of the current prevention and control program

CPCp = cost of the proposed prevention and control program

Deaths averted

ICER = (CPCp-CPCc)/(Dc-Dp)

Where: Dc = number of deaths under the current situation

Dp = number of deaths under the proposed program

DALYs averted

ICER = {(CPCp+COIp)-(CPCc+COIc)}/(DALYc-DALYp)

Where DALYc = number of disability-adjusted life years under the

current situation

DALYp = number of disability-adjusted life years under the

proposed program

One way sensitivity analysis:

- Discount rate of cost at 6% and no discount for the clinical outcomes
- Prices of vaccination (including acquisition cost, delivery cost, non-medical costs)
- Proportion of PEP administered via the ID route

Probabilistic sensitivity analysis (PSA) will employ the Monte Carlo simulation technique covering variability of parameters, i.e. discount rate, price of vaccine, dog vaccination coverage, proportion of PEP administered via ID or IM routes, ratio of dog: inhabitant, and geographical program coverage (e.g. targeting the whole country versus targeting high-risk areas).

**6. Results**

Based on current data, the costs of rabies prevention and control activities within the animal health sector in 2009 is estimated to be 51,161 million VND. These costs cover the cost of dog vaccination, surveillance, and management (IEC, training and general administration). The cost of illness associated with dog bites or rabies in 2009 is estimated to be 3,090,874 million VND which includes the cost of dog bite injuries, PEP, and cases of rabies. These projected costs during 2009-2018 will be 470,676 million VND and 38,564,903 million VND, respectively (Table 7). Parameters used in the analyses are presented in Table 8. Parameters with variability (dog population, costs, number of deaths, PEP, proportion of ID-PEP, and RIG) used for probabilistic sensitivity analysis are presented as type of distribution and standard error (SE). Further sensitivity analysis include best case (dog population =5 million, current dog vaccination=40%, proportion of ID-PEP=80%, proportion of RIG=20%, average IM-PEP/case=3 doses, average ID-PEP/ case=6 doses), worst case (GDP and income growth=0%, dog population =7 million, current dog vaccination=30%, proportion of ID-PEP=50%, proportion of RIG=50%, cost of Rabisin=5,000VND/10 doses, ID-PEP and RIG=50% of PEP, wastage rate of the vaccines=10%, cost of Verorab=148,000 VND/vial, cost f RIG=700,000VND/vial), scenario 1 (discount rate 6% and 3% for cost and DALYs, respectively) and scenario 2 (discount rate 3% and 0% for cost and DALYs, respectively). The reference case is estimated with parameters during the proposed project period are presented in Table 9 and the results are demonstrated in Table 10. It is shown that the proposed program results in a decrease in the burden of deaths and DALYs, whilst the cost is also less than that of the current program. For reference cases, the cost of prevention and control of the proposed program (number 17 in Table 10) is 931,160 million VND and the total cost of illness (number 18 in Table 10) is 11,497,989 million VND. The number of deaths (discounted to the year 2009) of the current program and proposed programs are 766 and 203, respectively. The number of DALYs (discounted to the year 2009) of the current program and proposed programs are 17,423 and 4,609, respectively. Regarding the cost-benefit analysis, the net present value in 2009 is 26,606,430 million VND. The proposed program creates life and DALYs averted with less cost comparing to those of the current program (minus values of ICER). In other words, the proposed program dominates the current program and similar results occur at all sensitivity analyses (Table 11).

Further analyses are focused on regions with high mortality rates (Table 4). The average annual mortality rate of the country was 1.12 per million population, most of the regions have rates less than 1, while those of the North East and Central Highlands were 5.09 and 2.28 per million population, respectively. Therefore, a simulation analysis is focused on these two regions. The population of these two regions is 17% of the total country population and the number of dogs is estimated based on the human population. The cost of the proposed program is estimated as 50% of the country because the proposed budget is composed of variable budget dependent on a number of provinces (indirect to population) and fixed costs of centralized activities. The cost of the current program for these two provinces is 79,785 and 6,441,590 million VND for prevention and control, and cost of illness, respectively (number 7, 8 of Table 12). The cost of the proposed program for these two provinces is 210,007 and 1,920,537 million VND for prevention and control, and cost of illness, respectively (number 71,1 8 of Table 12). The numbers of death and DALYs are decreased from 128 to 34 lives and 2,910 to 770 DALYs, respectively. The net present value is 4,390,831 VND and the proposed program also dominates the current program in all scenarios in all sensitivity analyses (Table 13).

**Table 7: Cost of the current program.**

|  |  | Total cost at 2009 prices (x1,000VND) | |
| --- | --- | --- | --- |
|  | COST | 2009 | 2009-2018 |
| 1 | Cost of management | 9,800,000 | 86,103,867 |
| 2 | Cost of surveillance (lab) | 18,238 | 169,576 |
| 3 | Cost of dog vaccination | 41,342,818 | 384,403,057 |
| 3.1 | Rabisin vaccine | 744,718 | 6,924,347 |
| 3.2 | Dog vaccine administration | 33,322,552 | 309,831,102 |
| 3.3 | Time & transportation of dog owner | 7,275,548 | 67,647,609 |
| 4 | Direct medical of dog bite injuries | 87,659,558 | 815,053,344 |
| 5 | Total cost of PEP | 1,822,284,634 | 16,943,493,829 |
| 5.1 | Verorab vaccine | 209,503,095 | 1,947,947,277 |
| 5.2 | RIG | 98,546,266 | 916,277,301 |
| 5.3 | Human vaccine administration | 507,456,680 | 4,718,301,946 |
| 5.4 | Time, transportation and meal of PEP case | 1,006,778,593 | 9,360,967,305 |
| 6 | Total cost of rabid case | 1,180,929,408 | 20,806,355,479 |
| 6.1 | Direct medical costs of human cases of rabies | 70,198 | 652,693 |
| 6.2 | Direct non-medical costs of human cases of rabies | 216,815 | 2,015,929 |
| 6.3 | Indirect cost of human cases of rabies | 1,180,642,395 | 20,803,686,857 |
|  |  |  |  |
|  | Total cost of prevention &control (1+2+3) | 51,161,056 | 470,676,500 |
|  | Total cost of illness (4+5+6) | 3,090,873,600 | 38,564,902,652 |

Table 8: Parameters used in the analyses.

|  | Parameter description | Reference case | SE | Distribution | Best case | Worst case | Scenario 1 | Scenario 2 |
| --- | --- | --- | --- | --- | --- | --- | --- | --- |
| 1 | Reference value |  |  |  |  |  |  |  |
| 1.1 | Population in 2008 | 86,210,800 | n/a | n/a | 86,210,800 | 86,210,800 | 86,210,800 | 86,210,800 |
| 1.2 | Average annual population growth rate (%) | 1.32 | n/a | n/a | 1.32 | 1.32 | 1.32 | 1.32 |
| 1.3 | GDP per capita in 2008 (x 1000VND) | 17,141 | n/a | n/a | 17,141 | 17,141 | 17,141 | 17,141 |
| 1.4 | Average annual growth of GDP per capita (current price)(%) | 0.14 | n/a | n/a | 0.14 | 0.00 | 0.14 | 0.14 |
| 1.5 | Daily income (x1000VND) | 88 | n/a | n/a | 88 | 88 | 88 | 88 |
| 1.6 | Average annual growth of income (%) | 18 | n/a | n/a | 18 | 0.00 | 18 | 18 |
| 1.7 | Cost discount rate (%) | 3.00 | n/a | n/a | 0.03 | 0.03 | 0.60 | 0.03 |
| 1.8 | Death/ DALYS discount rate (%) | 3.00 | n/a | n/a | 3.00 | 3.00 | 3.00 | 0.00 |
| 1.9 | Life expectancy (years) | 72 | n/a | n/a | 72 | 72 | 72 | 72 |
|  |  |  |  |  |  |  |  |  |
| 2 | Animal sector |  |  |  |  |  |  |  |
| 2.1 | Dog population in 2008 | 6,000,000 | 577,350 | gamma | 5,000,000 | 7,000,000 | 6,000,000 | 6,000,000 |
| 2.2 | Dog/inhabitant | 0.07 | 0.01 | gamma | 0.06 | 0.08 | 0.07 | 0.07 |
| 2.3 | Current dog vaccination coverage | 0.35 | n/a | n/a | 0.40 | 0.30 | 0.35 | 0.35 |
| 2.4 | Proposed dog vaccination coverage rate | 0.70 | n/a | n/a | 0.70 | 0.70 | 0.70 | 0.70 |
| 2.5 | Cost of Rabisin/10 dose vial (x1000VND) | 3.50 | n/a | n/a | 3.50 | 5.00 | 3.50 | 3.50 |
| 2.6 | Dog vaccine wastage rate | 0.00 | n/a | n/a | 0.00 | 0.10 | 0.00 | 0.00 |
| 2.7 | Dog vaccine administration cost (not including cost of vaccine) | 15.66 | 5.01 | gamma | 15.66 | 15.66 | 15.66 | 15.66 |
| 2.8 | Dog owner cost (travel, worktime) | 3.42 | 3.42 | gamma | 3.42 | 3.42 | 3.42 | 3.42 |
| 2.9 | Current investigation of dog specimen investigation rate | 0.00 | n/a | n/a | 0.00 | 0.00 | 0.00 | 0.00 |
| 2.1 | Proposed investigation of dog specimen investigation rate | 0.00 | n/a | n/a | 0.00 | 0.00 | 0.00 | 0.00 |
| 2.11 | Cost/investigation of dog specimen including transportation(x1000VND) | 450 | n/a | n/a | 450 | 450 | 450 | 450 |
| 3 | Human sector |  |  |  |  |  |  |  |
| 3.1 | Death/ million population | 0.94 | 0.11 | gamma | 0.94 | 0.94 | 0.94 | 0.94 |
| 3.2 | PEP case/ 1000 population | 7.08 | 0.28 | gamma | 7.08 | 7.08 | 7.08 | 7.08 |
| 3.3 | Probability of ID/ PEP case | 0.64 | 0.30 | beta | 0.80 | 0.50 | 0.64 | 0.64 |
| 3.4 | Proportion of RIG among PEP cases | 0.25 | 0.30 | beta | 0.20 | 0.50 | 0.25 | 0.25 |
| 3.5 | Average IM-PEP dose/ case | 5.00 | n/a | n/a | 3.00 | 5.00 | 5.00 | 5.00 |
| 3.6 | Average ID-PEP dose/ case | 8.00 | n/a | n/a | 6.00 | 8.00 | 8.00 | 8.00 |
| 3.7 | Average AIG dose/case | 1.00 | n/a | n/a | 1.00 | 1.00 | 1.00 | 1.00 |
| 3.8 | Average vaccine amount/ IM-PEP case (ml) | 2.50 | n/a | n/a | 1.50 | 2.50 | 2.50 | 2.50 |
| 3.9 | Average vaccine amount/ ID-PEP case (ml) | 0.80 | n/a | n/a | 0.60 | 0.80 | 0.80 | 0.80 |
| 3.1 | Vaccine vial/ IM-PEP case | 5.00 | n/a | n/a | 3.00 | 5.00 | 5.00 | 5.00 |
| 3.11 | Vaccine vial/ ID-PEP case | 1.60 | n/a | n/a | 1.20 | 1.60 | 1.60 | 1.60 |
| 3.12 | Wastage rate of ID_PEP | 0.00 | n/a | n/a | 0.00 | 0.10 | 0.00 | 0.00 |
| 3.13 | Wastage rate of IM_PEP | 0.00 | n/a | n/a | 0.00 | 0.10 | 0.00 | 0.00 |
| 3.14 | Wastage rate of RIG | 0.00 | n/a | n/a | 0.00 | 0.10 | 0.00 | 0.00 |
| 3.15 | Cost of Verorab/0.5 ml vial (x1000VND) | 120 | n/a | n/a | 120 | 148 | 120 | 120 |
| 3.16 | RIG cost/ single dose vial (x1000VND) | 650 | n/a | n/a | 650 | 700 | 650 | 650 |
| 3.17 | Vaccine administration cost/ dose (not include cost of vaccine)(x1000VND) | 114 | 40 | gamma | 114 | 114 | 114 | 114 |
| 3.18 | Direct non-medical+indirect cost of PEP/ case (x1000VND) | 1,627 | 192 | gamma | 1,627 | 1,627 | 1,627 | 1,627 |
| 3.19 | Direct medical cost of dog bite injuries/ case (x1000 VND) | 142 | 142 | gamma | 142 | 142 | 142 | 142 |
| 3.2 | Direct medical cost of rabid patient/ case (x1000VND) | 852 | 86 | gamma | 852 | 852 | 852 | 852 |
| 3.21 | Direct non-medical of rabid patient/ case (x1000VND) | 2,633 | 2,633 | gamma | 2,633 | 2,633 | 2,633 | 2,633 |
| 3.22 | Indirect cost of rabid patient/ case (x1000VND in 2009 prices) | 14,335,910 | n/a | n/a | 14,335,910 | 14,335,910 | 14,335,910 | 14,335,910 |
| 4 | Management |  |  |  |  |  |  |  |
| 4.1 | Current annual cost (x1000VND) | 9,800,000 | n/a | n/a | 9,800,000 | 9,800,000 | 9,800,000 | 9,800,000 |
| 4.2 | Proposed annual cost(x1000VND) | 18,044,248 | n/a | n/a | 18,044,248 | 18,044,248 | 18,044,248 | 18,044,248 |

Table 9: Estimated parameters.

| Year | 2008 | 2009 | 2010 | 2011 | 2012 | 2013 | 2014 | 2015 | 2016 | 2017 | 2018 |
| --- | --- | --- | --- | --- | --- | --- | --- | --- | --- | --- | --- |
| Human population | 86,210,800 | 87,350,698 | 88,505,669 | 89,675,910 | 90,861,625 | 92,063,018 | 93,280,295 | 94,513,668 | 95,763,349 | 97,029,553 | 98,312,500 |
| Dog population | 6,000,000 | 6,079,333 | 6,159,716 | 6,241,161 | 6,323,683 | 6,407,296 | 6,492,015 | 6,577,853 | 6,664,827 | 6,752,951 | 6,842,240 |
| CURRENT PROGRAM |  |  |  |  |  |  |  |  |  |  |  |
| Total number of vaccinated dog | 2,100,000 | 2,127,767 | 2,155,900 | 2,184,406 | 2,213,289 | 2,242,554 | 2,272,205 | 2,302,249 | 2,332,690 | 2,363,533 | 2,394,784 |
| Number of vaccine dose provided | 2,100,000 | 2,127,767 | 2,155,900 | 2,184,406 | 2,213,289 | 2,242,554 | 2,272,205 | 2,302,249 | 2,332,690 | 2,363,533 | 2,394,784 |
| Number of dog vaccine vial provided | 210,000 | 212,777 | 215,590 | 218,441 | 221,329 | 224,255 | 227,221 | 230,225 | 233,269 | 236,353 | 239,478 |
| Number of dog specimen | 40 | 41 | 41 | 42 | 42 | 43 | 43 | 44 | 44 | 45 | 46 |
| Number of injuries | 610,678 | 618,753 | 626,934 | 635,223 | 643,622 | 652,133 | 660,755 | 669,492 | 678,344 | 687,313 | 696,401 |
| Number of PEP cases | 610,678 | 618,753 | 626,934 | 635,223 | 643,622 | 652,133 | 660,755 | 669,492 | 678,344 | 687,313 | 696,401 |
| Number death | 81 | 82 | 83 | 85 | 86 | 87 | 88 | 89 | 90 | 91 | 93 |
| Number of ID-PEP case | 391,269 | 396,442 | 401,684 | 406,995 | 412,377 | 417,829 | 423,354 | 428,952 | 434,623 | 440,370 | 446,193 |
| Number of IM-PEP case | 219,409 | 222,310 | 225,250 | 228,228 | 231,246 | 234,303 | 237,401 | 240,540 | 243,721 | 246,943 | 250,208 |
| Number of RIG case (dose) | 149,631 | 151,610 | 153,614 | 155,645 | 157,703 | 159,789 | 161,901 | 164,042 | 166,211 | 168,409 | 170,635 |
|  |  |  |  |  |  |  |  |  |  |  |  |
| PROPOSED PROGRAM |  |  |  |  |  |  |  |  |  |  |  |
| Number of PEP case (%) | 100 | 100 | 50 | 50 | 50 | 50 | 25 | 25 | 25 | 25 | 25 |
| ID/ PEP case (%) | - | 64 | 75 | 75 | 75 | 75 | 75 | 75 | 75 | 75 | 75 |
| Number of death (%) | 100 | 100 | 75 | 50 | 25 | - | - | - | - | - | - |
| Number of vaccinated dog; attack phase | 4,200,000 | 4,255,533 | 4,311,801 | 4,368,813 | 4,426,578 | 4,485,107 | 4,544,410 | 4,604,497 | 4,665,379 | 4,727,066 | 4,789,568 |
| Total number of vaccinated dog | 4,200,000 | 4,255,533 | 4,311,801 | 4,368,813 | 4,426,578 | 4,485,107 | 4,544,410 | 4,604,497 | 4,665,379 | 4,727,066 | 4,789,568 |
| Number of vaccine dose provided | 4,200,000 | 4,255,533 | 4,311,801 | 4,368,813 | 4,426,578 | 4,485,107 | 4,544,410 | 4,604,497 | 4,665,379 | 4,727,066 | 4,789,568 |
| Number of dog vaccine vial provided | 420,000 | 425,553 | 431,180 | 436,881 | 442,658 | 448,511 | 454,441 | 460,450 | 466,538 | 472,707 | 478,957 |
| Number of dog specimen | 900 | 912 | 924 | 936 | 949 | 961 | 974 | 987 | 1,000 | 1,013 | 1,026 |
| Number of injuries | 610,678 | 618,753 | 626,934 | 635,223 | 643,622 | 652,133 | 660,755 | 669,492 | 678,344 | 687,313 | 696,401 |
| Number of PEP cases | 610,678 | 618,753 | 313,467 | 317,612 | 321,811 | 326,066 | 165,189 | 167,373 | 169,586 | 171,828 | 174,100 |
| Number death | 81 | 82 | 63 | 42 | 21 | - | - | - | - | - | - |
| Number of ID-PEP case | 391,269 | 396,442 | 200,842 | 203,498 | 206,188 | 208,915 | 105,838 | 107,238 | 108,656 | 110,092 | 111,548 |
| Number of IM-PEP case | 219,409 | 222,310 | 112,625 | 114,114 | 115,623 | 117,152 | 59,350 | 60,135 | 60,930 | 61,736 | 62,552 |
| Number of RIG case (dose) | 149,631 | 151,610 | 76,807 | 77,823 | 78,852 | 79,894 | 40,475 | 41,010 | 41,553 | 42,102 | 42,659 |

Table 10: Analysis results for the whole country (x million VND at 2009 prices).

|  |  | Reference case | Best case | Worst case | Scenario 1 | Scenario 2 |
| --- | --- | --- | --- | --- | --- | --- |
|  | CURRENT PROGRAM |  |  |  |  |  |
| 1 | Cost of management | 86,104 | 86,104 | 86,104 | 25,896 | 86,104 |
| 2 | Cost of surveillance (lab) | 170 | 141 | 141 | 57 | 170 |
| 3 | Cost of dog vaccination | 384,403 | 366,098 | 277,400 | 130,156 | 384,403 |
| 3.1 | Rabisin vaccine | 6,924 | 6,595 | 7,772 | 2,345 | 6,924 |
| 3.2 | Dog vaccine administration | 309,831 | 295,077 | 221,308 | 104,907 | 309,831 |
| 3.3 | Time & transportation of dog owner | 67,648 | 64,426 | 48,320 | 22,905 | 67,648 |
| 4 | Direct medical of dog bite injuries | 815,053 | 815,053 | 815,053 | 236,547 | 815,053 |
| 5 | Total cost of PEP | 16,943,494 | 14,872,463 | 19,104,693 | 4,917,390 | 16,943,494 |
| 5.1 | Verorab vaccine | 1,947,947 | 1,076,985 | 2,920,516 | 565,339 | 1,947,947 |
| 5.2 | RIG | 916,277 | 747,906 | 2,214,953 | 265,925 | 916,277 |
| 5.3 | Human vaccine administration | 4,718,302 | 3,686,605 | 4,608,256 | 1,369,359 | 4,718,302 |
| 5.4 | Time, transportation and meal of PEP case | 9,360,967 | 9,360,967 | 9,360,967 | 2,716,767 | 9,360,967 |
| 6 | Total cost of rabid case | 20,806,355 | 20,806,355 | 217,621 | 13,182 | 20,806,355 |
| 6.1 | Direct medical cost of human rabies case | 653 | 653 | 653 | 189 | 653 |
| 6.2 | Direct non-medical of human rabies case | 2,016 | 2,016 | 2,016 | 585 | 2,016 |
| 6.3 | Indirect cost of human rabies case | 20,803,687 | 20,803,687 | 214,952 | 12,407 | 20,803,687 |
|  |  |  |  |  |  |  |
| 7 | Total cost of prevention &control (1+2+3) | 470,677 | 452,343 | 363,645 | 156,109 | 470,677 |
| 8 | Total cost of illness (4+5+6) | 38,564,903 | 36,493,872 | 20,137,367 | 5,167,119 | 38,564,903 |
|  |  |  |  |  |  |  |
|  | OUTCOME |  |  |  |  |  |
| 9 | Number of death | 766 | 766 | 766 | 766 | 874 |
| 10 | Loss of DALYs | 17,423 | 17,423 | 17,423 | 17,423 | 37,126 |
|  |  |  |  |  |  |  |
|  | PROPOSED PROGRAM |  |  |  |  |  |
| 11 | Cost of management | 158,539 | 158,539 | 158,539 | 47,680 | 158,539 |
| 12 | Cost of surveillance (lab) | 3,815 | 3,180 | 3,180 | 1,292 | 3,815 |
| 13 | Cost of dog vaccination | 768,806 | 640,672 | 647,266 | 260,313 | 768,806 |
| 13.1 | Rabisin vaccine | 13,849 | 11,541 | 18,135 | 4,689 | 13,849 |
| 13.2 | Dog vaccine administration | 619,662 | 516,385 | 516,385 | 209,814 | 619,662 |
| 13.3 | Time & transportation of dog owner | 135,295 | 112,746 | 112,746 | 45,810 | 135,295 |
| 14 | Direct medical of dog bite injuries | 815,053 | 815,053 | 815,053 | 236,547 | 815,053 |
| 15 | Total cost of PEP | 7,351,862 | 6,453,232 | 8,289,617 | 3,256,211 | 7,351,862 |
| 15.1 | Verorab vaccine | 845,224 | 467,309 | 1,267,226 | 374,358 | 845,224 |
| 15.2 | RIG | 397,577 | 324,520 | 961,079 | 176,091 | 397,577 |
| 15.3 | Human vaccine administration | 2,047,294 | 1,599,635 | 1,999,544 | 906,766 | 2,047,294 |
| 15.4 | Time, transportation and meal of PEP case | 4,061,768 | 4,061,768 | 4,061,768 | 1,798,996 | 4,061,768 |
| 16 | Total cost of rabid case | 3,331,074 | 3,331,074 | 57,568 | 7,308 | 3,331,074 |
| 16.1 | Direct medical cost of human rabies case | 173 | 173 | 173 | 122 | 173 |
| 16.2 | Direct non-medical of human rabies case | 533 | 533 | 533 | 377 | 533 |
| 16.3 | Indirect cost of human rabies case | 3,330,368 | 3,330,368 | 56,862 | 6,809 | 3,330,368 |
|  |  |  |  |  |  |  |
| 17 | Total cost of prevention &control (11+12+13) | 931,160 | 802,390 | 808,985 | 309,285 | 931,160 |
| 18 | Total cost of illness (14+15+16) | 11,497,989 | 10,599,359 | 9,162,238 | 3,500,066 | 11,497,989 |
|  |  |  |  |  |  |  |
|  | OUTCOME |  |  |  |  |  |
| 19 | Number of death | 203 | 203 | 203 | 203 | 209 |
| 20 | Loss of DALYs | 4,609 | 4,609 | 4,609 | 4,609 | 8,859 |
|  |  |  |  |  |  |  |
|  | COST-BENEFIT ANALYSIS |  |  |  |  |  |
| 21 | Total cost of intervention (17-7) | 460,484 | 350,047 | 445,340 | 153,176 | 460,484 |
| 22 | Total benefit (8-18) | 27,066,913 | 25,894,513 | 10,975,129 | 1,667,053 | 27,066,913 |
| 23 | Net present value (NPV) (22-21) | 26,606,430 | 25,544,466 | 10,529,790 | 1,513,878 | 26,606,430 |
|  | Internal rate of return (IRR) (23/21*100) | 5,778 | 7,297 | 2,364 | 988 | 5,778 |
| 24 | Benefit-to-cost ratio (22/21) | 59 | 74 | 25 | 11 | 59 |
|  |  |  |  |  |  |  |
|  | COST-EFFECTIVENESS ANALYSIS |  |  |  |  |  |
| 25 | Total death averted (9-19) | 563 | 563 | 563 | 563 | 666 |
| 26 | Total DALYs averted (10-20) | 12,814 | 12,814 | 12,814 | 12,814 | 28,267 |
| 27 | ICER; death averted [(17+18-16.3)-(7+8-6.3)]/25 | -16,217 | -14,332 | -18,416 | -2,678 | -13,720 |
| 28 | ICER; DALYs averted [(17+18-16.3)-(7+8-6.3)]/26 | -713 | -630 | -809 | -118 | -323 |

Table 11: Probabilistic sensitivity analysis of the reference case of the whole country (x million VND at 2009 prices).

|  | Net present value | Benefit-to-cost ratio | ICER; death averted | ICER; DALYs averted |
| --- | --- | --- | --- | --- |
| Mean | 26,647,502 | 63 | -84,566 | -3,976 |
| SD | 2,563,292 | 18 | 3,358 | 148 |
| 95% CI |  |  |  |  |
| Upper level | 26,806,374 | 65 | -84,358 | -3,967 |
| Lower level | 26,488,631 | 62 | -84,774 | -3,986 |

Table 12: Analysis results for the high mortality rate region (x million VND at 2009 prices).

|  |  | Reference case | Best case | Worst case | Scenario 1 | Scenario 2 |
| --- | --- | --- | --- | --- | --- | --- |
|  | CURRENT PROGRAM |  |  |  |  |  |
| 1 | Cost of management | 14,570 | 14,570 | 14,570 | 4,382 | 14,570 |
| 2 | Cost of surveillance (lab) | 170 | 835 | 1,169 | 49 | 170 |
| 3 | Cost of dog vaccination | 65,046 | 366,098 | 388,360 | 18,878 | 65,046 |
| 3.1 | Rabisin vaccine | 1,172 | 6,595 | 10,881 | 340 | 1,172 |
| 3.2 | Dog vaccine administration | 52,427 | 295,077 | 309,831 | 15,216 | 52,427 |
| 3.3 | Time & transportation of dog owner | 11,447 | 64,426 | 67,648 | 3,322 | 11,447 |
| 4 | Direct medical of dog bite injuries | 136,140 | 136,140 | 136,140 | 39,511 | 136,140 |
| 5 | Total cost of PEP | 2,830,113 | 2,484,184 | 3,191,103 | 821,364 | 2,830,113 |
| 5.1 | Verorab vaccine | 325,370 | 179,891 | 487,821 | 94,430 | 325,370 |
| 5.2 | RIG | 153,048 | 124,925 | 369,969 | 44,418 | 153,048 |
| 5.3 | Human vaccine administration | 788,109 | 615,783 | 769,728 | 228,727 | 788,109 |
| 5.4 | Time, transportation and meal of PEP case | 1,563,585 | 1,563,585 | 1,563,585 | 453,788 | 1,563,585 |
| 6 | Total cost of rabid case | 3,475,336 | 3,475,336 | 36,350 | 2,202 | 3,475,336 |
| 6.1 | Direct medical cost of human rabies case | 109 | 109 | 109 | 32 | 109 |
| 6.2 | Direct non-medical of human rabies case | 337 | 337 | 337 | 98 | 337 |
| 6.3 | Indirect cost of human rabies case | 3,474,891 | 3,474,891 | 35,904 | 2,072 | 3,474,891 |
|  |  |  |  |  |  |  |
| 7 | Total cost of prevention &control (1+2+3) | 79,785 | 381,503 | 404,099 | 23,309 | 79,785 |
| 8 | Total cost of illness (4+5+6) | 6,441,590 | 6,095,660 | 3,363,594 | 863,076 | 6,441,590 |
|  |  |  |  |  |  |  |
|  | OUTCOME |  |  |  |  |  |
| 9 | Number of death | 128 | 128 | 128 | 128 | 146 |
| 10 | Loss of DALYs | 2,910 | 2,910 | 2,910 | 2,910 | 6,201 |
|  |  |  |  |  |  |  |
|  | PROPOSED PROGRAM |  |  |  |  |  |
| 11 | Cost of management | 79,269 | 79,269 | 79,269 | 23,840 | 79,269 |
| 12 | Cost of surveillance (lab) | 646 | 3,180 | 4,451 | 187 | 646 |
| 13 | Cost of dog vaccination | 130,092 | 640,672 | 906,173 | 37,756 | 130,092 |
| 13.1 | Rabisin vaccine | 2,343 | 11,541 | 25,389 | 680 | 2,343 |
| 13.2 | Dog vaccine administration | 104,855 | 516,385 | 722,939 | 30,431 | 104,855 |
| 13.3 | Time & transportation of dog owner | 22,894 | 112,746 | 157,844 | 6,644 | 22,894 |
| 14 | Direct medical of dog bite injuries | 136,140 | 136,140 | 136,140 | 39,511 | 136,140 |
| 15 | Total cost of PEP | 1,227,999 | 1,077,899 | 1,384,635 | 543,893 | 1,227,999 |
| 15.1 | Verorab vaccine | 141,180 | 78,056 | 211,668 | 62,530 | 141,180 |
| 15.2 | RIG | 66,408 | 54,205 | 160,531 | 29,413 | 66,408 |
| 15.3 | Human vaccine administration | 341,964 | 267,191 | 333,989 | 151,459 | 341,964 |
| 15.4 | Time, transportation and meal of PEP case | 678,447 | 678,447 | 678,447 | 300,491 | 678,447 |
| 16 | Total cost of rabid case | 556,397 | 556,397 | 9,616 | 1,221 | 556,397 |
| 16.1 | Direct medical cost of human rabies case | 29 | 29 | 29 | 20 | 29 |
| 16.2 | Direct non-medical of human rabies case | 89 | 89 | 89 | 63 | 89 |
| 16.3 | Indirect cost of human rabies case | 556,279 | 556,279 | 9,498 | 1,137 | 556,279 |
|  |  |  |  |  |  |  |
| 17 | Total cost of prevention &control (11+12+13) | 210,007 | 723,121 | 989,894 | 61,783 | 210,007 |
| 18 | Total cost of illness (14+15+16) | 1,920,537 | 1,770,437 | 1,530,391 | 584,625 | 1,920,537 |
|  |  |  |  |  |  |  |
|  | OUTCOME |  |  |  |  |  |
| 19 | Number of death | 34 | 34 | 34 | 34 | 35 |
| 20 | Loss of DALYs | 770 | 770 | 770 | 770 | 1,480 |
|  |  |  |  |  |  |  |
|  | COST-BENEFIT ANALYSIS |  |  |  |  |  |
| 21 | Total cost of intervention (17-7) | 130,221 | 341,618 | 585,795 | 38,474 | 130,221 |
| 22 | Total benefit (8-18) | 4,521,053 | 4,325,224 | 1,833,203 | 278,452 | 4,521,053 |
| 23 | Net present value (NPV) (22-21) | 4,390,831 | 3,983,606 | 1,247,408 | 239,978 | 4,390,831 |
|  | Internal rate of return (IRR) (23/21*100) | 3,372 | 1,166 | 213 | 624 | 3,372 |
| 24 | Benefit-to-cost ratio (22/21) | 35 | 13 | 3 | 7 | 35 |
|  |  |  |  |  |  |  |
|  | COST-EFFECTIVENESS ANALYSIS |  |  |  |  |  |
| 25 | Total death averted (9-19) | 94 | 94 | 94 | 94 | 111 |
| 26 | Total DALYs averted (10-20) | 2,140 | 2,140 | 2,140 | 2,140 | 4,721 |
| 27 | ICER; death averted [(17+18-16.3)-(7+8-6.3)]/25 | -15,651 | -11,321 | -12,980 | -2,541 | -13,240 |
| 28 | ICER; DALYs averted [(17+18-16.3)-(7+8-6.3)]/26 | -688 | -498 | -570 | -112 | -312 |

Table 13: Probabilistic sensitivity analysis of reference case of the high mortality rate region (x million VND at 2009 prices).

|  | Net present value | Benefit-to-cost ratio | ICER; death averted | ICER; DALYs averted |
| --- | --- | --- | --- | --- |
| Mean | 4,393,482 | 50 | -83,888 | -3,947 |
| SD | 429,845 | 20 | 3,494 | 154 |
| 95% CI |  |  |  |  |
| Upper level | 4,420,124 | 51 | -83,672 | -3,937 |
| Lower level | 4,366,841 | 48 | -84,105 | -3,956 |

**4. CONCLUSIONS AND RECOMMENDATIONS**

An economic evaluation of rabies prevention and control activities has been designed based on the proposed 10-year rabies prevention and control program, i.e. an increase in dog vaccination coverage level to 70%, a reduction in the costs associated with PEP, and improvement in disease surveillance. The framework is composed of cost-effectiveness and cost-benefits analyses, in relation to deaths and DALYs averted. Local and international experts were consulted during data collection, not only in relation to collection of national statistics, but also for the estimation of various epidemiological parameters and the effectiveness of proposed interventions, including necessary assumptions. Articles published in peer-reviewed journals also provided useful reference information. This study found that the proposed program creates a net benefit and dominates the current program in terms of deaths and DALYs averted in all scenarios and both at the country and region levels.

This study employs modelling approaches with assumptions particularly costs of the proposed program. However, the Excel-based software has been developed for the study and therefore, the study can be applied to other regions and provinces with specific data on costs of the proposed program.

The findings provide valuable guidance to policy-makers in relation to allocation of funding and resources towards rabies prevention and control in Vietnam, as well as serving as a practical example of rabies advocacy to ASEAN Plus 3 countries.

**5. ACKNOWLEDGEMENTS**

I am extremely grateful to Dr. Anna Dean for organising and supporting every aspect of the consultancy and Prof. Dinh Kim Xuyen from the National Rabies Control Program, Prof. Nguyen Thi Hong Hanh from NIHE, Dr. Francois-Xavier Meslin from WHO Headquarters, Dr. Bee Lee Ong from WHO Regional Office for the Western Pacific, and Dr. Katie Hampson (WHO International Consultant in March 2009) for their very helpful discussions and provision of data. Many thanks are also due to Dr. Nguyen Thi Thi Tho and Dr. Le Phuong Mai from NIHE and Ms. Vu Thuy Duong from Hanoi University of Pharmacy for their support in data collection and interpretation. I am also very grateful for the participation of the respondents of interviews and questionnaires. I really appreciated the assistance of staff from the animal health offices and preventive medicine centres in Thai Nguyen city, Dien Hoa district in Thai Nguyen province, NIHE, Tu Liem district in Hanoi, St. Paul Hospital, and the National Institute of Infectious and Tropical Diseases.

**REFERENCES**
