# Supplementary material for: Estimating the economic impact of canine rabies to Viet Nam 2005–2014
Source: PLoS Negl Trop Dis. 2018 Oct 11;12(10):e0006866. doi: 10.1371/journal.pntd.0006866 (PMC6199002; doi:10.1371/journal.pntd.0006866)
Supplement: S2 Text — (DOCX) [file pntd.0006866.s002.docx]

*Table 1 : Rabies in Dogs, 2008-2015*

| Năm/ YEAR | Số tỉnh/ No of infected provinces | Số huyện/ No of infected districts | Số xã  No of infected communes | Sốchóchết và tiêu hủy  No of dead dogs and culled due to rabies |
| --- | --- | --- | --- | --- |
| 2008 | 5 | 7 | 28 | 110 |
| 2009 | 2 | 4 | 8 | 25 |
| 2010 | 8 | 14 | 42 | 150 |
| 2011 | 5 | 6 | 11 | 58 |
| 2012 | 8 | 19 | 34 | 268 |
| 2013 | 10 | 20 | 27 | 260 |
| 2014 | 23 | 53 | 65 | 125 |
| 2015 | 27 | 52 | 63 | 85 |

*Table 2: Vaccination in dogs*

| *Năm/ Year* | *Tổng đàn chó*  *Dog population (head)* | *Tỷ lệ tiêm phòng*  *Vaccination coverage (%)* |
| --- | --- | --- |
| 2011 | 8,585,856 | 37,79% |
| 2012 | 8.437.861 | 38,20 |
| 2013 | 8.239.877 | 44,22 |
| 2014 | 8.195.809 | 46,98 |
| 2015 | 8.427.525 | 30,39 |

Source: DAH 2015
